# Hyperuricemia, gout and the associated comorbidities in China: findings from a prospective study of 0.5 million adults

**DOI:** 10.1016/j.lanwpc.2025.101572

**Published:** 2025-05-14

**Authors:** Pek Kei Im, Christiana Kartsonaki, Maria G. Kakkoura, Olaa Mohamed-Ahmed, Ling Yang, Yiping Chen, Huaidong Du, Xiaoming Yang, Hua Zhang, Dianjianyi Sun, Canqing Yu, Jun Lv, Liming Li, Zhengming Chen, Iona Y. Millwood, China Kadoorie Biobank Collaborative Group, Junshi Chen, Zhengming Chen, Robert Clarke, Rory Collins, Liming Li, Jun Lv, Richard Peto, Robin Walters, Daniel Avery, Maxim Barnard, Derrick Bennett, Ruth Boxall, Ka Hung Chan, Yiping Chen, Zhengming Chen, Charlotte Clarke, Jonathan Clarke, Robert Clarke, Huaidong Du, Ahmed Edris Mohamed, Hannah Fry, Simon Gilbert, Pek Kei Im, Andri Iona, Maria Kakkoura, Christiana Kartsonaki, Kshitij Kolhe, Hubert Lam, Kuang Lin, James Liu, Mohsen Mazidi, Iona Millwood, Sam Morris, Qunhua Nie, Alfred Pozarickij, Maryam Rahmati, Paul Ryder, Dan Schmidt, Becky Stevens, Iain Turnbull, Robin Walters, Baihan Wang, Lin Wang, Neil Wright, Ling Yang, Xiaoming Yang, Pang Yao, Xiao Han, Can Hou, Qingmei Xia, Chao Liu, Jun Lv, Pei Pei, Dianjianyi Sun, Canqing Yu, Lang Pan, Zengchang Pang, Ruqin Gao, Shanpeng Li, Haiping Duan, Shaojie Wang, Yongmei Liu, Ranran Du, Liang Cheng, Xiaocao Tian, Hua Zhang, Dan Hu, Xiaoyan Zheng, Yujie Wang, Wei Sun, Shichun Yan, Xiaoming Cui, Chi Wang, Zhenyuan Wu, Lishun Zhai, Zhaoxi Pang, Shiwen Dong, Huiming Luo, Jinyan Chen, Bin He, Dingwei Sun, Xingren Wang, Tingting Ou, Xiangyang Zheng, Dewei Zheng, Shuai Yang, Yilei Li, Lihui Li, Xingjiao Chen, Jinyi Zhou, Ran Tao, Jian Su, Xikang Fan, Zongming Cheng, Yuxiao Huang, Yan Lu, Yujie Hua, Li Xing, Shuxian Wang, Jianrong Jin, Juping Ma, Jinchao Liu, Kaifei Zhu, Hongfu Ren, Xingfeng Shen, Ge Zhong, Wei Mao, Zhenzhen Lu, Ling He, Lifang Zhou, Changping Xie, Jian Lan, Tingping Zhu, Jinxue Tan, Liuping Wei, Liyuan Zhou, Sisi Wang, Xianping Wu, Ningmei Zhang, Xiaofang Chen, Xiaoyu Chang, Zhuo Wang, Yujin He, Mingqiang Yuan, Xia Wu, Xiaofang Chen, Zhaodong Wang, Qiang Sun, Yang Lin, Faqing Chen, Xiaolan Ren, Lijun Chang, Feiming Zhong, Jianjun Feng, Weijie Hu, Xiaofang Zhang, Yalin Chen, Fei Wang, Jun Wang, Linqi Diao, Wanshen Guo, Zhiwei Han, Dongyang Zhao, Dengjun Zhu, Kai Kang, Shixian Feng, Huizi Tian, Yali Yan, Bing Han, Li Gao, Shaofang Li, Huafei Feng, Wei Tang, Xiaolin Li, Huarong Sun, Xiaocong Zhao, Ying Li, Chen Hu, Pan He, Xukui Zhang, Yuanyuan Jin, Hesheng Zhang, Min Yu, Ruying Hu, Hao Wang, Weiwei Gong, Jieming Zhong, Meng Wang, Chunxiao Xu, Keqing Gong, Hao Xu, Yuan Cao, Kaixu Xie, Lingli Chen, Xiaomei Tu, Chen Chen, Xiaojun Li, Li Yin, Huilin Liu, Yuan Liu, Yi Liu, Lei Yin, Xian Xie, Jing Wang, Bo Xiao, Pingsheng Lou, Yuan Peng, Libo Zhang, Chan Qu, Qili Jiang, Yanling Chen, Yan Zhao

**Affiliations:** a Clinical Trial Service Unit and Epidemiological Studies Unit (CTSU), Nuffield Department of Population Health, University of Oxford, Oxford, UK; b NCDs Prevention and Control Department, Qingdao CDC, Qingdao, Shandong, China; c Department of Epidemiology and Biostatistics, School of Public Health, Peking University Health Science Center, Beijing, China; d Peking University Center for Public Health and Epidemic Preparedness & Response, Beijing, China; e Key Laboratory of Epidemiology of Major Diseases (Peking University), Ministry of Education, Beijing, China

**Keywords:** Gout, Comorbidity, China, Prospective study, Hyperuricemia

## Abstract

**Background:**

Despite the growing prevalence of hyperuricemia and gout, their epidemiology and associated comorbidity burden remains poorly studied in many populations, including China. We aimed to examine the patterns of plasma urate level, prevalence of hyperuricemia, and incidence of gout, and investigate the associations of gout with a range of comorbidities and all-cause mortality in Chinese adults.

**Methods:**

The prospective China Kadoorie Biobank recruited 512,724 adults aged 30–79 years from ten diverse areas in 2004–2008 and measured plasma urate level among 16,817 participants. The incidence of gout and other diseases and deaths were monitored by electronic linkages with registries and hospital records. Cox and logistic regression yielded adjusted HRs and ORs for risks of mortality and comorbidities associated with gout, hyperuricemia, and urate level.

**Findings:**

The gout incidence rate was 23.4 per 100,000 person-years, and was higher in men and older participants, and varied substantially by region. Gout was associated with higher risks of all-cause mortality (HR = 1.58, 95% CI 1.37–1.82), CVD (1.87, 1.64–2.14), CKD (5.61, 4.45–7.07), urolithiasis (2.50, 1.85–3.38), diabetes (1.99, 1.51–2.62), diseases of the oesophagus, stomach, and duodenum (2.14, 1.72–2.66), infectious and parasitic diseases (1.91, 1.47–2.48), arthropathies (6.06, 4.98–7.38), and other musculoskeletal disorders (2.10, 1.77–2.51). Most of these associations were bi-directional, sustained over time and little affected by adjustment for cardiometabolic risk factors. Moreover, participants who developed gout were more likely to have multiple major diseases and more hospitalisations. Among the subset with plasma urate measured, 15% had hyperuricemia, which was more common in men, older women, and urban residents, and was associated with increased risks of gout, all-cause mortality, and several cardiometabolic, renal, digestive, and musculoskeletal diseases.

**Interpretation:**

In Chinese adults, gout was associated with several comorbidities and a poor health trajectory. Our findings reinforce the need for prevention and management of gout and associated comorbidities.

**Funding:**

10.13039/501100017647Kadoorie Charitable Foundation, 10.13039/501100001809National Natural Science Foundation of China, Noncommunicable Chronic Diseases-National Science and Technology Major Project, 10.13039/501100000274British Heart Foundation, 10.13039/501100000289Cancer Research UK, 10.13039/100010269Wellcome Trust, 10.13039/501100000265UK Medical Research Council, 10.13039/501100024811Nuffield Department of Population Health at the University of Oxford.

**Research in context**

**Evidence before this study**

Gout is the most common form of inflammatory arthritis with increasing prevalence globally. Understanding the epidemiology and comorbidity relationships of gout may improve our knowledge on the burden of gout and its potential shared pathogenesis with other diseases.

We searched PubMed for reports on the prospective associations of gout with disease outcomes published up to 1 November 2024, using the terms: “gout” AND “disease OR comorbidit∗ OR incidence OR risk OR mortality OR death”. Most previous studies were based primarily on routine health record data in Western or high-income populations (e.g. UK Clinical Practice Research Datalink [CPRD], and several national medical record or insurance databases) with limited information on lifestyle risk factors, and had focused on a specific disease or disease category. A few prospective cohort studies (e.g. the Framingham Study, the Health Professionals Follow-up Study) were identified, which were primarily based on Western populations and largely limited to CVD. Overall, previous studies consistently reported positive associations of gout with CVD and renal diseases. Only a few studies, including those based on the UK CPRD and Swedish healthcare registers, have examined the prospective associations of gout with a range of comorbidity and mortality outcomes. These studies have reported positive associations of gout with several major diseases beyond CVD and renal diseases, including genitourinary, metabolic/endocrine, musculoskeletal, and liver diseases, and deaths from infection and digestive diseases. Some of these associations (e.g. diabetes, musculoskeletal diseases) were reported by other studies, however for other diseases evidence was limited and inconsistent. Evidence from well-designed prospective cohort studies which allow for comprehensive adjustments for potential biases, and from other populations, is limited.

**Added value of this study**

In this large prospective study of 0.5 million Chinese adults with 12-year follow-up of linked hospital records and comprehensive information on lifestyle risk factors, we examined the patterns of incidence of gout, the prevalence of its precursor hyperuricemia, and levels of plasma urate by socio-demographic groups, and comprehensively assessed the prospective associations of gout with a range of major morbidity outcomes and all-cause mortality.

Among Chinese adults, gout and hyperuricemia were more common in men, urban residents, and older participants, with substantial geographical variation across ten study regions in China. As well as confirming previous known associations of gout with cardiovascular and renal diseases and urolithiasis, we also found associations for several other diseases (e.g. diabetes, arthropathies, and diseases of the oesophagus, stomach, and duodenum) less established to be related to gout previously. Most of these associations were bi-directional, and were not fully explained by shared risk factors or reverse causation. Hyperuricemia also showed similar associations with a range of cardiometabolic, renal, digestive, and musculoskeletal disease outcomes. Overall, individuals who developed gout were more likely to have multiple comorbidities, more hospitalisations, and higher mortality risks.

**Implications of all the available evidence**

Evidence in Western and Chinese populations shows that people with gout had increased risks of a range of major diseases beyond CVD and chronic renal disease, with an overall high comorbidity burden and poorer health trajectory. This knowledge can inform and reinforce prevention and management strategies of gout and associated comorbidities. Findings on the sex-, age-, and geographical variation in hyperuricemia prevalence and gout incidence rates may inform screening and prevention strategies in China. Prevention and management guidelines for hyperuricemia and gout may consider inclusion of assessment and monitoring of a wider range of comorbidities. Future genetic and multi-omics research are warranted to investigate the potential causal relevance and underlying mechanisms linking gout to different major diseases.

## Introduction

Gout is the most common form of inflammatory arthritis affecting nearly 56 million people worldwide in 2020, with the number of cases projected to rise to 96 million in 2050 primarily driven by population growth and ageing.^1^ Gout is characterised by the deposition of monosodium urate (MSU) crystals in and around joints, which triggers painful acute inflammatory arthritis episodes (gout flares) that may become recurrent and progress to, when untreated, chronic gout causing joint damage.^2^ Hyperuricemia, the sustained elevated serum urate concentration that facilitates the formation of MSU crystals, is the most important risk factor for gout.^2^ The burden of gout varies substantially across regions with the highest prevalence in high-income North America and the Pacific regions,^1^^,^^3^ potentially reflecting differences in genetic and lifestyle risk factors. In China, nationwide surveys reported an increase in the prevalence of gout from <1% to 3% between 1995 and 2019^4^ and of hyperuricemia from 11% to 14% between 2015 and 2019,^5^ which may be attributed to adverse changes in dietary patterns (e.g. alcohol, purine-rich food, and sugar-sweetened beverage intake) and an increase in obesity and metabolic syndrome that are suggested to be risk factors for hyperuricemia and gout.^2^

Despite the high population burden and decreased quality of life related to gout, gout is generally poorly managed with low uptake and adherence to urate-lowering therapy.^2^ Furthermore, gout has been associated with cardiovascular diseases (CVDs) and chronic kidney disease (CKD),^6^, ^7^, ^8^, ^9^ potentially exacerbating the related public health burden, especially in ageing populations. The cause–effect relationships and underlying mechanisms of these observed associations, and the relationships between gout and other conditions, are poorly understood. Large-scale population-based epidemiological studies offer an opportunity to investigate the occurrence patterns and associated comorbidities of gout.^10^ However, existing evidence has mostly involved routine register databases in high-income Western populations with limited adjustments for lifestyle risk factors^10^, ^11^, ^12^ or epidemiological surveys based on self-reported comorbidities in high-risk populations (e.g. in the Pacific Islands).^3^ Large-scale investigations on the prospective associations between gout and different diseases, especially in other populations, are sparse. Understanding the epidemiology of gout and hyperuricemia and their associated comorbidities in a Chinese population, whose environmental and lifestyle exposures, metabolic and genetic profile, and disease patterns vary substantially from Western populations,^13^ will improve knowledge of the burden of gout and potential shared aetiology and pathogenesis with other diseases. This may inform prevention and management approaches in China and elsewhere.

This study characterised the descriptive epidemiology and comorbidity relationships of gout in 0.5 million adults from the prospective China Kadoorie Biobank (CKB) study. We aimed to: (1) examine the level of plasma urate, prevalence of hyperuricemia, and incidence rate of gout by socio-demographic groups (sex, age, education) and regions; and (2) assess the associations of gout with a range of major morbidities and all-cause mortality, overall and among population subgroups.

## Methods

### Study population

Details of the study design and methods of the CKB, and its wide applications in epidemiological investigations of the determinants of chronic diseases, have been previously reported.^13^, ^14^, ^15^, ^16^ Briefly, 512,724 adults aged 30–79 years were recruited from ten geographically diverse (five rural, five urban) areas across China during 2004–2008. The ten study areas were selected to cover a diverse range of socio-economic development, risk factor exposures, and disease patterns (see Appendix p7-p8). Potentially eligible participants without major disabilities were identified through official residential records in 100–150 administrative units (rural villages and urban residential committees) within each study area. At local study assessment clinics, trained health workers administered a laptop-based questionnaire recording socio-demographic factors, lifestyle factors (e.g. alcohol drinking, smoking, physical activity, and diet), medical history (self-rated health and a range of doctor-diagnosed health conditions), and female reproductive factors. The questionnaire had built-in checks to identify and minimise missing items, data entry errors, and inconsistencies. Physical measurements (e.g. blood pressure and anthropometry) were undertaken using calibrated instruments and standard protocols. A 10 ml non-fasting blood sample was collected for long-term storage and onsite tests including random blood glucose level (Johnson & Johnson SureStep Plus Meter), with time since last meal recorded. Three resurveys of ∼5% randomly selected surviving participants were subsequently conducted in 2008, 2013–2014, and 2021–2022 using similar procedures.

### Follow-up for mortality and morbidity

The vital status of participants was obtained periodically from local death registries, supplemented by annual active confirmation through local residential, health insurance, and administrative records. Additional information on morbidity since baseline was collected through linkage with established disease registries (for cancer, stroke, ischaemic heart disease [IHD], and diabetes) and the national health insurance system, which records any episodes of hospitalization and almost has universal coverage of the study areas. All events were coded with International Classification of Diseases, 10th revision (ICD-10) codes, blinded to the baseline information. The main exposure of this study was clinically diagnosed gout reported from electronic health records during follow-up (predominantly captured via inpatient hospitalisations [see Appendix p9]; prior gout was not assessed in the baseline medical history questionnaire), defined as the first event with an ICD-10 code M10. The total number of gout episodes reported over follow-up was also recorded.

The disease outcomes for this report were selected based on a combination of previous evidence in relation to gout,^11^^,^^12^ major causes of mortality and morbidity,^17^ and statistical power. These disease incidence outcomes, identified through electronic linkage records, included: CVDs, including IHD and stroke separately; any cancer; CKD; urolithiasis; diabetes; chronic obstructive pulmonary disease (COPD); liver disease; diseases of the oesophagus, stomach, and duodenum; disorders of the gallbladder, biliary tract, and pancreas; infectious and parasitic diseases; arthropathies (excluding gout); other musculoskeletal disorders; other autoimmune diseases; fracture; and all-cause mortality (see Appendix p10 for disease definitions and ICD-10 codes, and Appendix p11 for number of recorded events). By 1 January 2019, 56,550 (11%) participants had died, and only 4028 (<1%) were lost to follow-up.

### Measurement of plasma urate level

In a nested case–control study of CVD, we measured a range of biomarkers, including urate, using baseline plasma samples from ∼18,000 participants, at the Oxford NDPH Wolfson Laboratory (Appendix p4). Overall 16,817 participants had urate level measurements (μmol/L), and hyperuricemia was defined as urate level >360 μmol/L for both men and women based on recommended levels for long-term control.^18^

### Statistical analysis

The main analyses of gout included the whole study cohort. For analyses involving the urate subset, inverse probability of sampling weights were applied to account for the nested case–control study design (Appendix p4, p6, p12).

Means and percentages of baseline characteristics were calculated by gout status, adjusted for sex, age (in 10-year intervals), and the ten study areas, as appropriate. Linear regression models were used to estimate adjusted mean levels of urate and prevalence of hyperuricemia by socio-demographic factors (age, sex, study area, education level), adjusted for sex, age, study area, and fasting time before blood sample collection, as appropriate. Additional adjustments for creatinine (as a measure of kidney function), alcohol consumption (for men), and menopausal status (for women) were made to examine differences in the levels of urate and hyperuricemia by baseline age. The incidence rates (95% confidence intervals [CIs], calculated using normal approximation) of gout were estimated as the number of gout cases per 100,000 person-years (py) overall and by socio-demographic factors, standardized by sex, age-at-risk, and study area of the CKB study population, as appropriate.

Cox regression models with a time-updated exposure for gout, counting individuals with incident gout as exposed from their time of diagnosis, were used to estimate hazard ratios (HRs) and 95% CIs for incident disease outcomes and all-cause mortality between ages 35 and 84 years. As age is an important determinant of gout and multiple diseases, age was used as the underlying time scale. Participants contributed time at risk from their age at baseline or 35 years (whichever later), and were followed up until the first occurrence of the corresponding disease endpoint, or were censored at the earliest of the following events: death from other causes, lost to follow-up, or reaching the general censoring date of 1 January 2019 or age 84 years. The models were stratified by sex and the ten study areas, and were adjusted for baseline age, education, alcohol consumption, smoking, physical activity, and dietary factors (fish or seafood, red meat, poultry, soybean, dairy products, and fresh fruits). Participants with relevant self-reported prior diseases were excluded from the analyses (Appendix p10). Similar Cox models were used to estimate HRs associated with duration since gout diagnosis (non-exposed [reference group], 0–2 years, 2–5 years, 5+ years), a time-updated exposure defined as the time interval between gout diagnosis and time at risk for the disease outcome, with test for trend assessed among gout patients by fitting gout duration as an ordinal variable. Associations of shorter duration since gout diagnosis (non-exposed [reference group], 0–1 year, 1–2 years, 2+ years), and the number of reported gout episodes (none [reference group], 1 episode, 2+ episodes), with disease risks were also assessed (Appendix p5). Confounding variables were selected based on a priori knowledge of standard risk factors and demonstrated associations with urate level, gout, and major disease outcomes.

To assess the associations of preceding major diseases (prevalent and incident cases, see Appendix p10), and duration since their diagnosis, with subsequent risk of gout, Cox models were used with time-updated exposure for preceding disease to estimate adjusted HRs for gout (see Appendix p5 for detailed methods).

The associations were examined separately by sex, and for selected major outcomes (CVD, CKD, diabetes, arthropathies, all-cause mortality) by index age, study area, and education level, with chi-squared tests for heterogeneity applied to the log_e_ HRs and their standard errors. Sensitivity analyses were performed by: additional adjustments for BMI and systolic blood pressure (SBP) to assess independent associations beyond shared cardiometabolic risk factors; excluding individuals with prior CVD, CKD, or poor self-rated health at baseline; adding a one-year lag to the time-updated exposure for gout to minimise reverse causation and potential impact of concomitant diagnoses. The associations of urate level and hyperuricemia with major disease outcomes were assessed using logistic regression models (Appendix p6).

Logistic regression models were used to estimate odds ratios (ORs) for having more than one comorbidity throughout the study period, comparing gout patients vs. other participants, among those without any self-reported prior major diseases (see Appendix p4 for definition). To assess the cumulative morbidity burden, the total number of hospitalizations were estimated for participants who had gout (treated as a time-updated group) vs. those not, using the mean cumulative count of hospital episodes.^19^

The proportional hazards assumption was assessed using plots of scaled Schoenfeld residuals and the associated chi square tests (no clear evidence of violation). For analyses involving more than two exposure categories, floating standard errors based on quasi-variances were used to estimate group-specific 95% CIs for all categories (including the reference group) to facilitates comparisons between any two categories.^20^, ^21^, ^22^ For comparisons of two groups (i.e. an exposure category with the reference group), conventional 95% CIs were reported. The Benjamini-Hochberg method was used to account for multiple comparisons within each stream of analyses. All *P*-values were two-sided. Statistical significance (at the 5% level) was evaluated using both false discovery rate (FDR)-adjusted and conventional *P*-values. All analyses used R software (version 4.4.1).

### Ethics approval

The CKB complies with all the required ethical standards for medical research on human subjects. Ethical approval was obtained from the Ethical Review Committee of the Chinese Centre for Disease Control and Prevention (Beijing, China, 2004; Reference: 005/2004) and the Oxford Tropical Research Ethics Committee, University of Oxford (UK, 2005; Reference: 025-04). All participants provided written informed consent.

### Role of the Funding source

The funders had no role in the study design, data collection, formal data analysis and interpretation, writing of the manuscript, or the decision to submit the article for publication.

## Results

Among the 512,724 study participants (Table 1), the mean age at baseline was 52 (SD 10.7) years, 41% were men and 56% lived in rural areas. During a median of 12.1 (interquartile range [IQR]: 11.1, 13.1) years of follow-up, 1402 participants (1071 men, 331 women) developed gout, corresponding to an incidence rate of 23.4 (43.2 among men, 9.6 among women) per 100,000 person-years. The median age at first diagnosis was 67.6 (IQR 59.8, 75.6) years and among those with gout, 25% (28% men vs. 15% women) had two or more recorded gout episodes over the follow-up period (Appendix p13).

**Table 1:** Baseline characteristics of study participants, by gout status.

Characteristics	Overall	Non-cases	Gout cases
	(n = 512,724)	(n = 511,322)	(n = 1402)
**Demographic and lifestyle factors**			
Mean age, years (SD)	52.0 (10.7)	52.0 (10.7)	57.2 (10.5)
Women, %	59.0	59.1	24.9
Urban, %	44.1	44.1	48.6
Education >6 years (i.e. primary school or above), %	49.2	49.2	45.2
Household income >20,000 yuan^a^/year, %	42.7	42.7	44.9
Current alcohol drinkers, %	14.9	14.8	21.1
Men	33.3	33.2	41.4
Women	2.1	2.1	2.0
Mean alcohol intake^b^, g/week (SD)	271.7 (242.4)	271.4 (242.2)	328 (266.0)
Men	285.7 (245.3)	285.3 (245.1)	341.4 (266.0)
Women	115.6 (127.4)	115.5 (127.2)	197.5 (231.5)
Current smokers, %	26.4	26.5	19.7
Men	61.1	61.1	54.5
Women	2.4	2.4	1.8
Daily tea drinkers, %	26.1	26.1	23.7
Men	40.8	40.8	38.6
Women	15.9	15.9	16.8
Physical activity, mean MET-h/d (SD)	21.1 (13.9)	21.1 (13.9)	19.7 (11.9)
**Anthropometry**, mean (SD)			
Body mass index, kg/m^2^	23.7 (3.4)	23.7 (3.4)	25.1 (3.3)
Systolic blood pressure, mmHg	131.1 (21.3)	131.1 (21.3)	138.2 (22.4)
Random glucose, mmol/Litre	5.9 (1.9)	5.9 (1.9)	6.0 (2.1)
**Medical history and health status**^c^, %			
Poor self-rated health	10.4	10.3	13.8
Coronary heart disease	3.0	3.0	5.2
Stroke or transient ischaemic attack	1.7	1.7	2.2
Liver cirrhosis or hepatitis	1.2	1.2	0.8
Emphysema or chronic bronchitis	2.6	2.6	2.3
Cancer	0.5	0.5	0.1
Peptic ulcer	3.9	3.9	4.8
Gallstone or gallbladder disease	6.0	6.0	8.5
Kidney disease	1.5	1.5	2.7
Rheumatoid arthritis	2.1	2.1	4.8
Prevalent diabetes	5.9	5.9	5.7
**Frequent dietary consumption**^d^, %			
Red meat	47.2	47.2	48.6
Weekly poultry	28.2	28.2	30.5
Fish or seafood	8.9	8.9	8.9
Fresh fruits	28.2	28.2	28.6
Fresh vegetables	98.3	98.3	98.4
Soybean products	9.9	9.9	8.9
Dairy products	11.9	11.9	10.6
Preserved vegetables	22.6	22.6	22.7
Spicy food	30.1	30.1	31.8

Prevalences and means were adjusted for sex, age (10-year groups), and ten study areas, where appropriate, using linear regression.

MET-h/d, metabolic equivalent of task per hour per day; SD, standard deviation.

a At the exchange rate as of February 2025, 1 yuan is approximately equal to 0.14 U S. dollars.

b Weekly alcohol intake was calculated among current drinkers only.

c Medical history and self-rated health status were self-reported at baseline, except for prevalent diabetes which included both self-reported and screen-detected diabetes.

d Frequent dietary consumption means 4+ days per week unless otherwise specified.

Participants who developed gout were more likely to be men, be older at baseline, live in urban areas, and have less education than those without gout (Table 1). They also had higher BMI and SBP and poorer self-rated health, were less physically active, and among men were more likely to be current alcohol drinkers but less likely to be current smokers.

### Mean urate level, prevalence of hyperuricemia, and gout incidence rate patterns

Among the subset of 16,817 participants with urate measurements (Appendix p14), the mean urate level was 279.2 (SD 83.1) μmol/L (304.7 μmol/L in men, 241.3 μmol/L in women), with 15.4% (21.4% men, 5.3% women) defined as having hyperuricemia (Appendix p15). Both urate level (HR = 2.99, 95% CI 2.53–3.53, per SD increment) and hyperuricemia (11.31, 6.30–20.33) were strongly associated with increased risk of gout (Appendix p16).

**Fig. 1:**
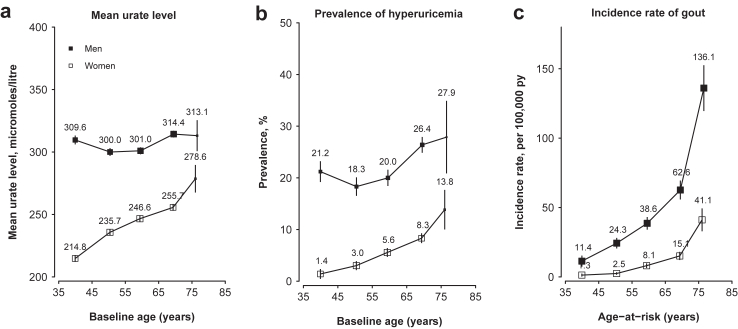
**Age-specific mean urate level, hyperuricemia prevalence, and incidence rate of gout, by sex**. Adjusted mean urate level (a) and prevalence of hyperuricemia (b) were estimated using linear regression adjusted for study area and fasting time, with inverse probability weighting applied to account for the nested case–control study design. Incidence rates (c) were standardised by study area to the CKB study population. Each solid square represents the adjusted mean, prevalence, or incidence rate with the area inversely proportional to the variance of the estimate. The error bars indicate 95% CIs. CI, confidence interval; py, person-years.

Mean urate level and proportion of hyperuricemia generally increased with age, but this pattern differed by sex. Among men there were U-shaped relationships with higher mean urate level and proportion of hyperuricemia in age groups <45 years and >65 years, whereas among women these values increased steadily with age (Fig. 1a and b). The associations with age were unaltered with further adjustment for alcohol consumption in men, but were attenuated after adjustment for menopausal status in women and creatinine level in both sexes (Appendix p17). In contrast, incidence rates of gout increased continuously with age overall (range 5.3–78.4 per 100,000 py, <45 to 75+ years), and in men (11.4–136.1 per 100,000 py) and women (1.3–41.1 per 100,000 py), with greater absolute increases after age 65 years (Fig. 1c).

The incidence rate of gout was generally higher in urban than rural areas (Appendix p15, p18) and varied substantially across the ten study sites (∼1 in Henan to >50 in Hunan and Liuzhou, per 100,000 py), with broadly similar regional distribution patterns for urate level and hyperuricemia (except for two coastal cities Qingdao and Haikou which had considerably high prevalence of hyperuricemia) (Appendix p19, p20). Mean urate level and hyperuricemia prevalence, but not gout incidence, also tended to increase with education level (Appendix p15).

### Associations of gout with **risks of** incident morbidities and all-cause mortality

**Fig. 2:**
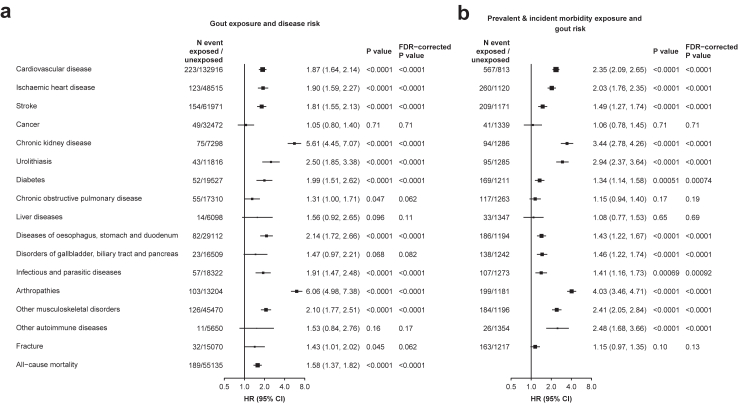
**Bi-directional associations between gout and major diseases**. Cox models were stratified by sex and study areas and were adjusted for baseline age, education, smoking, alcohol, physical activity, fish intake, red meat intake, poultry intake, soybean intake, dairy intake, and fresh fruit intake. (a) Displays adjusted HRs for risks of major diseases associated with gout, after excluding relevant prior diseases of interest. (b) Displays adjusted HRs for risk of gout associated with preceding prevalent (where recorded at baseline) and incident major diseases. FDR-adjusted *P* values were applied to correct for multiple testing within (a) and (b), respectively. Each solid square represents HR with the area inversely proportional to the variance of the log HR. The horizontal lines indicate 95% CIs. HR, hazard ratio; CI, confidence interval; FDR, false discovery rate.

Gout was associated with increased risks of incident CVD (adjusted HR = 1.87, 95% CI 1.64–2.14), including both IHD (1.90, 1.59–2.27) and stroke (1.81, 1.55–2.13), diabetes (1.99, 1.51–2.62), and urolithiasis (2.50, 1.85–3.38), with an HR of 5.61 (95% CI 4.45–7.07) for CKD (Fig. 2a). There were also significant positive associations between gout and diseases of the oesophagus, stomach, and duodenum (2.14, 1.72–2.66), infectious and parasitic diseases (1.91, 1.47–2.48), arthropathies (6.06, 4.98–7.38), and other musculoskeletal disorders (2.10, 1.77–2.51). There was suggestive evidence for elevated risks of COPD (1.31, 1.00–1.71) and fracture (1.43, 1.01–2.02), but the associations were non-significant after correction for multiple comparisons. Overall, gout patients had an HR of 1.58 (95% CI 1.37–1.82) for all-cause mortality when compared with participants who did not develop gout.

The excess risks for most of these diseases associated with gout generally persisted for up to 5+ years following gout diagnosis, except for urolithiasis, COPD, and infectious and parasitic diseases (with few cases) for which the associations were attenuated to the null after 5 years (Table 2; Appendix p21, p23). The associations for IHD, CKD, and particularly arthropathies appeared to be weaker over time since gout diagnosis but tests for trend were not significant. Having two or more hospitalised gout episodes was associated with stronger elevated risks, especially for CKD (Appendix p22).

**Table 2:** Number of events and adjusted HRs for specific diseases associated with duration of gout.

Major diseases	No gout	Duration of gout (years)				FDR-corrected P for trend
		0–2	2–5	5+	P for trend	
Cardiovascular disease						
N	132,916	122	74	27		
HR (95% CI)	1.00 (0.99–1.01)	2.09 (1.75–2.49)	1.63 (1.30–2.05)	1.78 (1.22–2.60)	0.64	0.84
Ischaemic heart disease						
N	48,515	62	45	16		
HR (95% CI)	1.00 (0.98–1.02)	2.09 (1.63–2.68)	1.83 (1.37–2.45)	1.54 (0.94–2.51)	0.65	0.84
Stroke						
N	61,971	78	51	25		
HR (95% CI)	1.00 (0.98–1.02)	1.95 (1.56–2.43)	1.58 (1.20–2.07)	2.00 (1.35–2.96)	0.47	0.81
Cancer						
N	32,472	26	15	8		
HR (95% CI)	1.00 (0.98–1.02)	1.20 (0.82–1.76)	0.87 (0.53–1.45)	1.05 (0.53–2.11)	0.16	0.42
Chronic kidney disease						
N	7298	38	26	11		
HR (95% CI)	1.00 (0.96–1.05)	6.44 (4.69–8.86)	5.23 (3.56–7.69)	4.39 (2.43–7.94)	0.29	0.62
Urolithiasis						
N	11,816	29	11	3		
HR (95% CI)	1.00 (0.97–1.04)	3.58 (2.49–5.15)	1.73 (0.96–3.13)	1.09 (0.35–3.37)	0.053	0.42
Diabetes						
N	19,527	26	16	10		
HR (95% CI)	1.00 (0.97–1.03)	2.15 (1.47–3.16)	1.68 (1.03–2.75)	2.21 (1.19–4.11)	0.82	0.87
Chronic obstructive pulmonary disease						
N	17,310	21	28	6		
HR (95% CI)	1.00 (0.97–1.03)	1.04 (0.68–1.59)	1.78 (1.23–2.58)	0.99 (0.44–2.21)	0.43	0.81
Liver diseases						
N	6098	9	4	1		
HR (95% CI)	1.00 (0.95–1.05)	2.17 (1.13–4.17)	1.25 (0.47–3.32)	0.62 (0.09–4.44)	>0.99	>0.99
Diseases of oesophagus, stomach and duodenum						
N	29,112	33	37	12		
HR (95% CI)	1.00 (0.98–1.02)	1.85 (1.31–2.60)	2.61 (1.89–3.60)	1.93 (1.09–3.39)	0.14	0.42
Disorders of gallbladder, biliary tract and pancreas						
N	16,509	9	10	4		
HR (95% CI)	1.00 (0.96–1.04)	1.24 (0.64–2.38)	1.72 (0.93–3.21)	1.53 (0.57–4.07)	0.75	0.85
Infectious and parasitic diseases						
N	18,322	30	22	5		
HR (95% CI)	1.00 (0.97–1.03)	2.14 (1.50–3.07)	1.99 (1.31–3.02)	1.04 (0.43–2.50)	0.59	0.84
Arthropathies						
N	13,204	61	34	8		
HR (95% CI)	1.00 (0.97–1.03)	7.65 (5.95–9.83)	5.40 (3.86–7.56)	2.93 (1.47–5.87)	0.11	0.42
Other musculoskeletal disorders						
N	45,470	67	40	19		
HR (95% CI)	1.00 (0.98–1.02)	2.46 (1.94–3.13)	1.81 (1.33–2.47)	1.79 (1.14–2.81)	0.71	0.85
Other autoimmune diseases						
N	5650	5	4	2		
HR (95% CI)	1.00 (0.95–1.06)	1.55 (0.65–3.73)	1.51 (0.56–4.01)	1.50 (0.37–6.01)	0.14	0.42
Fracture						
N	15,070	13	12	7		
HR (95% CI)	1.00 (0.97–1.03)	1.26 (0.73–2.16)	1.46 (0.83–2.56)	1.83 (0.87–3.84)	0.16	0.42
All-cause mortality						
N	55,135	76	84	29		
HR (95% CI)	1.00 (0.98–1.02)	1.37 (1.09–1.72)	1.88 (1.52–2.33)	1.47 (1.02–2.12)	0.17	0.42

Cox models were stratified by sex and study areas and were adjusted for baseline age, education, smoking, alcohol, physical activity, fish intake, red meat intake, poultry intake, soybean intake, dairy intake, and fresh fruit intake. HRs were presented with group-specific 95% CIs to enable comparison between any two groups. P for trend was obtained from fitting duration categories as ordinal variable in the Cox model among gout patients. HR, hazard ratio; CI, confidence interval. HR, hazard ratio; FDR, false discovery rate.

The associations of gout with liver diseases (1.56, 0.92–2.65), disorders of the gallbladder, biliary tract, and pancreas (1.47, 0.97–2.21), and other autoimmune diseases (1.53, 0.84–2.76) tended to be positive but were not statistically significant, and there was no evidence for association with cancer (1.05, 0.80–1.40) (Fig. 2a).

### Associations of preceding major diseases with risk of gout

Similar relationships between preceding major diseases and subsequent incidence of gout were observed. The risk of gout was elevated in participants with preceding CVD, CKD, urolithiasis, diabetes, diseases of the oesophagus, stomach, and duodenum, infectious and parasitic diseases, arthropathies, or other musculoskeletal disorders. The HRs for gout ranged from 1.34 (95% CI 1.14–1.58) for preceding diabetes to 4.03 (3.46–4.71) for arthropathies (Fig. 2b). The associations generally persisted over time since disease diagnosis, except for infectious and parasitic disease (Appendix p23). In addition, preceding disorders of the gallbladder, biliary tract, and pancreas (1.46, 1.22–1.74), and other autoimmune diseases (2.48, 1.68–3.66) were also associated with increased gout risk (Fig. 2b).

### Subgroup and sensitivity analyses

**Fig. 3:**
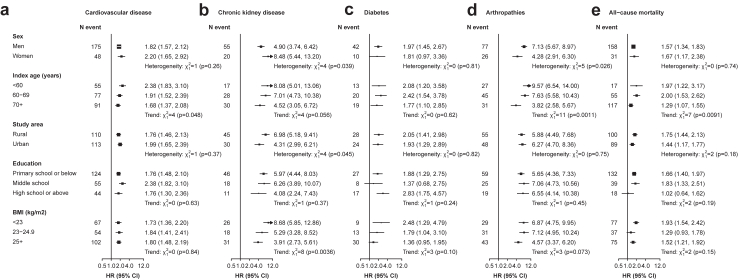
**Associations of gout with risks of selected incident major diseases and all-cause mortality, among subgroups**. Cox models were stratified by sex and study areas and adjusted for baseline age, education, smoking, alcohol, physical activity, fish intake, red meat intake, poultry intake, soybean intake, dairy intake, and fresh fruit intake. Each solid square represents HR with the area inversely proportional to the variance of the log HR. The horizontal lines indicate 95% CIs. HR, hazard ratio; CI, confidence interval.

The associations of gout with risks of selected major outcomes were generally stronger in participants with younger index age but broadly consistent across other socio-demographic subgroups, except for apparently stronger associations for CKD in women (P-heterogeneity = 0.039) and those with lower BMI (P-trend = 0.004), and for arthropathies in men (P-heterogeneity = 0.026) (Fig. 3, Appendix p24).

Additional adjustment for BMI and SBP slightly attenuated the associations of gout with CVD, CKD, urolithiasis, diabetes, and arthropathies (Appendix p25). Further excluding participants with prior CVD, CKD, or poor baseline self-rated health, or adding a one-year lag to gout exposure did not materially alter the findings.

Both urate level and hyperuricemia were positively associated with most of the studied outcomes in the subset, except for potentially non-linear inverse association with cancer and a non-significant weak inverse association with COPD (Appendix p26-p28). There were no clear patterns of associations of urate level with diseases of the oesophagus, stomach, and duodenum, infectious and parasitic diseases, or other autoimmune diseases.

### Multiple comorbidit**ies** and hospitalisations

Compared with participants who did not develop gout, those with gout were more likely to have a diagnosis of more than one other major disease, prior to or after their gout diagnosis, with higher ORs for increasing numbers of co-morbidities (ORs 4.11 [95% CI 3.56–4.74] for 2+ co-morbidities; 6.42 [4.63–8.91] for 5+ co-morbidities) (Appendix p29). Gout patients also had a poorer survival rate and higher numbers of hospitalizations for any causes, in particular cardiovascular, genitourinary, and musculoskeletal diseases, and for most other categories apart from cancers, and these excess hospitalisations further increased with increasing age-at-risk (Appendix p30).

## Discussion

Our study provides a comprehensive assessment of the epidemiology of hyperuricemia and gout in a large Chinese adult population. Among Chinese adults, hyperuricemia and gout were more common in men, urban residents, and older populations, with substantial geographical variation in gout incidence. Gout was positively associated with CVD, CKD, urolithiasis, diabetes, diseases of the oesophagus, stomach, and duodenum, infectious and parasitic diseases, and musculoskeletal disorders, for which the observed associations were bi-directional, generally consistent with associations observed for hyperuricemia (with a few exceptions), and sustained over time since gout diagnosis and after adjustment for cardiometabolic risk factors. Gout patients were more likely to have multiple major diseases, and had substantially more episodes of hospitalisation and higher mortality risk than those who did not develop gout.

The incidence rate of gout in our study was lower than that from the Global Burden of Disease (GBD) study for China in 1990–2017 (23.4 in CKB vs. 79.9–84.8 per 100,000 py),^23^ which may reflect differences in the underlying study population (e.g. regions, healthy volunteer effect in CKB), reporting sources, and analytical methodology (e.g. inpatient hospitalisations in CKB vs. modelling with various data input sources in GBD). Similarly, our observed median age at gout diagnosis was more advanced than previously reported in Western populations,^24^^,^^25^ which may be attributed to a number of cohort-specific factors including hospitalisation-based gout cases, healthy volunteer effect, and potentially our relatively lean study population given obesity has been associated with younger age of gout onset.^25^ Consistent with previous studies in China^5^^,^^26^, ^27^, ^28^ and elsewhere,^1^ we observed a higher occurrence of hyperuricemia and gout in men and in older participants, with a striking increase in urate levels and hyperuricemia with age in women but a U-shaped pattern in men. The sex- and age-differences may be explained by biological and lifestyle factors, such as reduced renal urate clearance related to declined kidney function with older age,^29^ changes in female hormones following menopause particularly reductions in estrogen,^30^ and potentially specific lifestyle patterns in younger men, although adjustment for alcohol consumption did not alter the observed male U-shaped pattern. Moreover, the increase in gout incidence with age in both sexes, consistent with previous studies,^27^^,^^28^ suggests the potential role of other age-related factors in influencing the development of gout compared to hyperuricemia. Our observed urban-rural difference and geographical variation in hyperuricemia and gout occurrence, with high-risk regions broadly overlapping with previous reports,^4^^,^^28^ may reflect the considerable regional differences within China of a combination of potential risk factors including economic development, lifestyle patterns (e.g. alcohol, meat, and seafood intake), metabolic syndrome, and potentially other (e.g. environmental) factors that may trigger gout flares and gout-related hospitalisations.^2^^,^^13^ For example, Liuzhou had a high proportion of frequent red meat and poultry consumers, whereas Qingdao and Haikou had considerably high proportion of regular beer drinkers and seafood consumers, respectively (Appendix 8). Further research is warranted to clarify the causal relevance of lifestyle and environmental risk factors on urate level and risk of gout which may inform public health strategies.

The links between gout and CVD,^6^^,^^8^^,^^9^^,^^31^ CKD,^7^^,^^11^^,^^32^ and urolithiasis^7^^,^^11^ have been consistently reported, mainly from observational studies in Western populations, or routine health record linkage studies with limited information and adjustments for lifestyle risk factors (e.g. diet, physical activity). Evidence from prospective cohort studies in other populations and particularly for renal disease is however limited. Using CKB, we demonstrated positive, bi-directional associations of gout with CVD, CKD, and urolithiasis in a Chinese population. Our risk estimates (HR = 1.87 for CVD, 5.61 for CKD, 2.50 for urolithiasis) were somewhat higher than previous reports from the UK Clinical Practice Research Datalink (CPRD) (HR = 1.58 for CVD,^6^ 3.18 for renal disease, 1.26 for urolithiasis^11^) and the Taiwan Longitudinal Health Insurance Database (1.34 for CHD,^9^ 1.57 for end-stage renal disease^32^), which may reflect between-study differences such as the severity of gout cases in CKB (nearly all of which were hospitalised) vs. primary care records,^6^^,^^9^ kidney disease definitions, and the risk factor profiles of the underlying study populations. We additionally found that the excess CKD risk increased with number of gout episodes, suggesting the potential importance of timely gout management. In contrast to previous reports,^6^^,^^33^ we did not find a stronger association between gout and CVD risk in women than men, which might have reflected the higher prevalence of certain shared risk factors (e.g. alcohol consumption) in Chinese men than women.^13^ Instead, we observed differential associations for CKD by sex, BMI, and potentially rural residency, suggesting potential effect modification by sex-specific factors, adiposity, and urban-rural differences in chronic disease management, however these subgroup analyses were based on small case numbers and should be interpreted with caution.

As in a few previous studies, we found positive associations of gout with risks of diabetes and musculoskeletal conditions. Our risk estimates appeared somewhat greater (adjusted HRs range 2–6 for diabetes and musculoskeletal conditions in CKB vs. up to ∼1.6 in other studies^11^^,^^34^), which may be due in part to the severity of our hospitalised gout cases. Previous evidence on the bi-directional relationships between gout and diabetes was mixed, with studies in Western and Singapore Chinese populations reporting a positive association between gout and subsequent risk of type 2 diabetes, but null or inverse associations of prior diabetes with gout risk.^11^^,^^35^ In CKB we observed positive bi-directional associations between gout and diabetes. We also found strong positive bi-directional associations of gout with arthropathies (mainly arthrosis and rheumatoid arthritis, see Appendix p11) and other musculoskeletal disorders, supporting previous findings for osteoarthritis and rheumatological disease from the UK CPRD^11^ and osteoporosis in a meta-analysis of four routine record studies.^34^ Existing evidence for other diseases was limited and inconsistent.^11^^,^^12^^,^^34^^,^^36^ A Swedish healthcare register study reported excess mortality risks from digestive and infectious diseases in gout patients,^12^ whereas a UK CPRD study found an increased risk of liver disease but not peptic ulcer disease.^11^ While not directly comparable with these studies due to heterogeneity in disease definitions, our study suggested potential positive associations between gout and digestive diseases particularly those of the oesophagus, stomach, and duodenum, and short-to medium-term risks of infectious and parasitic diseases; however, no clear associations were observed for urate level. Further investigations to clarify these associations and causal relevance, especially for infectious and gastrointestinal disorders previously less known to be associated with gout, are warranted. Previous findings on the association of gout with cancer was mixed with potential heterogeneity by cancer sites,^11^^,^^12^^,^^36^ and we found no clear relationship with overall cancer in CKB.

Several possible explanations exist for the associations between gout and comorbidities. The associations may be partly explained by shared lifestyle and metabolic risk factors, especially for cardiometabolic-renal diseases and arthropathies where further adjustments for BMI and SBP partly attenuated the associations. While concomitant diagnosis of comorbidities, especially CVD and CKD, detected through subsequent medical assessment and screening in gout patients (and vice versa) is possible, this was unlikely to explain fully most of our observed associations which were sustained over several years after initial gout diagnosis. In addition, gout and associated comorbidities may have shared or related aetiological mechanisms, contributing to short-term and long-term pathogenesis and disease risks. A key proposed mechanism linking gout to CVD and CKD is via hyperuricemia-induced effects, including endothelial dysfunction, renin-angiotensin system activation, and vascular and tubulointerstitial damage.^37^^,^^38^ However, Mendelian randomisation studies predominantly involving European-ancestry populations did not support a causal role of urate on clinical cardiometabolic-renal outcomes,^39^ but suggested potential genetic pleiotropic effects affecting both urate and metabolic traits.^40^ Other potential mechanisms include the formation of uric acid stones and other kidney stone types due to lower urinary pH,^41^ the presence of MSU crystals (e.g. in cartilage and joints, and potentially coronary arteries) and the associated inflammatory effects,^37^^,^^38^ and the use of gout flare medications (e.g. anti-inflammatory drugs, allopurinol, colchicine, corticosteroids),^33^^,^^37^ which may affect a range of health conditions.

To our knowledge, this is the among the first prospective cohort studies to investigate the relationships between gout and a range of different diseases with comprehensive adjustments for potential confounders in a Chinese population. Our replication of the well-known associations with CVD, CKD, and musculoskeletal disorders from previous studies provided validity as the positive control for our findings. However our study also had several limitations. First, our gout cases were identified mainly from hospitalisations. Milder gout cases not requiring hospitalisation are likely to be missed, leading to potential underestimation of gout incidence. Furthermore, some of our hospitalised gout cases might reflect recurrent gout rather than the time at first gout attack. Selection bias may also exist as hospitalised gout patients may have poorer general health and be more likely to be hospitalised more often for other diseases, potentially biasing our observed associations towards more severe and comorbid gout cases. Nevertheless, our findings on hyperuricemia generally supported most of the observed disease associations with gout, and the associations between hospitalised gout and comorbidities highlight important public health implications in both prevention and management of gout. Second, we did not have access to medication prescription records to identify gout patients, nor to account for medication for gout treatment, which may have beneficial or adverse effects on risks and progression of different comorbidities.^33^^,^^37^ Third, we lacked statistical power to assess the associations with other potentially related (e.g. neurodegenerative^2^) diseases and longer-term effects of gout (e.g. 10+ years duration), and period-specific HRs should be interpreted with caution due to potential built-in selection bias.^42^ Fourth, CKB was not designed to be nationally representative. Nonetheless, given large size and diversity of CKB and the comparable lifestyle and health status patterns with those reported in national representative surveys,^43^, ^44^, ^45^, ^46^ our findings should still be generalizable to the Chinese population at large. Finally, although we had carefully accounted for potential confounding effect by multiple lifestyle risk factors (e.g. alcohol drinking, smoking, diet; with good data agreement with objective measurements^47^ and known disease associations reported^14^^,^^48^), residual confounding may remain and we could not infer causality based on observational analyses. Future studies using multi-state models to analyse the transition between gout and comorbidities and identify multimorbidity clusters, and using genetic and multi-omics approaches to elucidate causal relevance and underlying mechanisms are warranted.

This study presents a comprehensive investigation of the distribution patterns of gout and hyperuricemia and the associated comorbidity burden in Chinese adults. Gout was associated with a range of comorbidities, some well-established and some less known, and increased hospitalisations and mortality risk. Our findings may inform public health strategies at local and national levels to strengthen the prevention and management of gout, including assessment and monitoring of associated comorbidities.

## Contributors

PKI, IYM, and ZC contributed to the conception of this paper. PKI, IYM, CK, and ZC planned the statistical analysis. CK, MK, OMA, and PKI contributed to the development of the methodology. PKI analysed the data and drafted the manuscript. PKI, IYM, CK, and ZC contributed to the interpretation of the results and the revision of manuscript. LL and ZC designed the study. LL, ZC, IYM, LY, YC, HD, XY, HZ, DS, CY, and JL contributed to data acquisition and general study management. XY provided administrative and technical support. PKI and IYM have accessed and verified the data used for these analyses. All authors critically reviewed the manuscript, and shared the final responsibility for the decision to submit for publication.

## Data sharing statement

The China Kadoorie Biobank (CKB) is a global resource for the investigation of lifestyle, environmental, blood biochemical and genetic factors as determinants of common diseases. The CKB study group is committed to making the cohort data available to the scientific community in China, the UK and worldwide to advance knowledge about the causes, prevention and treatment of disease. For detailed information on what data is currently available to open access users and how to apply for it, visit: https://www.ckbiobank.org/data-access. Researchers who are interested in obtaining the raw data from the China Kadoorie Biobank study that underlines this paper should contact ckbaccess@ndph.ox.ac.uk. A research proposal will be requested to ensure that any analysis is performed by bona fide researchers and - where data is not currently available to open access researchers - is restricted to the topic covered in this paper.

## Declaration of interests

The authors declare that they have no competing interests.
