# Supplementary material for: Hyperuricemia, gout and the associated comorbidities in China: findings from a prospective study of 0.5 million adults
Source: Lancet Reg Health West Pac. 2025 May 14;58:101572. doi: 10.1016/j.lanwpc.2025.101572 (PMC12167516; doi:10.1016/j.lanwpc.2025.101572)
Supplement: Appendix [file mmc1.pdf]

Supplementary material

Hyperuricemia, gout and the associated comorbidities in China: findings from a prospective study of 0.5 million adults

Pek Kei Im et al

Content List of Supplementary Material

Members of the China Kadoorie Biobank collaborative group 3

Supplementary Methods 4

Figure S1. Map of the ten study sites of CKB 7

Table S1. Baseline characteristics of study participants by ten study sites of CKB 8

Table S2. Distribution of gout incidents (n=1402) in CKB by reporting source 9

Table S3. Definitions of major diseases 10

Table S4. Number of disease events recorded via self-reported medical history at baseline and electronic health records over follow-up 11

Table S5. Number and weights for cases and controls in the subset of participants with urate measurement 12

Table S6. Adjusted ORs for having 2+ recorded gout episodes associated with socio-demographic factors, in 1402 gout patients 13

Table S7. Baseline characteristics of study participants with urate measurement 14

Table S8. Mean urate level, proportion of hyperuricemia, and incidence rate of gout, overall and by demographic groups 15

Table S9. Associations of urate level and hyperuricemia with risk of gout 16

Figure S2. Age-specific mean urate level, hyperuricemia prevalence, and incidence rate of gout, by sex, with further adjustments 17

Figure S3. Age-specific mean urate level, hyperuricemia prevalence, and incidence rate of gout, by sex and study area 18

Figure S4. Regional distribution of mean urate level, prevalence of hyperuricemia and incidence rate of gout, overall and by sex 19

Figure S5. Comparison of prevalence of hyperuricemia using sex-specific cut-off and common cut-off 20

Figure S6. Associations of short-term gout duration with risks of major diseases and all-cause mortality 21

Figure S7. Associations of number of gout episodes with risks of subsequent major diseases and all-cause mortality 22

Figure S8. Associations of gout duration with risks of major diseases, and of preceding disease duration with subsequent gout risk 23

Figure S9. Bi-directional associations of gout with major diseases, by sex 24

Figure S10. Sensitivity analyses of the associations of gout with risks of subsequent major diseases and all-cause mortality 25

Figure S11. Adjusted ORs for major diseases and all-cause mortality associated with urate level and hyperuricemia 26

Figure S12. Dose-response associations of urate level with major diseases and all-cause mortality 27

Figure S13. Adjusted ORs for major diseases and all-cause mortality associated with urate level and hyperuricemia, based on robust standard errors where applicable 28

Figure S14. Adjusted ORs for having multiple comorbidities associated with gout, overall and by sex....................29

Figure S15. Total expected hospitalisations overall and by major disease categories, and Kaplan-Meier curves for overall survival, from age-at-risk of 35 years, by gout status....................30

References....................31

Members of the China Kadoorie Biobank collaborative group

International Steering Committee: Junshi Chen, Zhengming Chen (PI), Robert Clarke, Rory Collins, Liming Li (PI), Jun Lv, Richard Peto, Robin Walters.

International Co-ordinating Centre, Oxford: Daniel Avery, Maxim Barnard, Derrick Bennett, Ruth Boxall, Ka Hung Chan, Yiping Chen, Zhengming Chen, Charlotte Clarke, Jonathan Clarke, Robert Clarke, Huaidong Du, Ahmed Edris Mohamed, Hannah Fry, Simon Gilbert, Pek Kei Im, Andri Iona, Maria Kakkoura, Christiana Kartsonaki, Kshitij Kolhe, Hubert Lam, Kuang Lin, James Liu, Mohsen Mazidi, Iona Millwood, Sam Morris, Qunhua Nie, Alfred Pozarickij, Maryam Rahmati, Paul Ryder, Dan Schmidt, Becky Stevens, Iain Turnbull, Robin Walters, Baihan Wang, Lin Wang, Neil Wright, Ling Yang, Xiaoming Yang, Pang Yao.

National Co-ordinating Centre, Beijing: Xiao Han, Can Hou, Qingmei Xia, Chao Liu, Jun Lv, Pei Pei, Dianjianyi Sun, Canqing Yu, Lang Pan.

10 Regional Co-ordinating Centres:

Qingdao CDC: Zengchang Pang, Ruqin Gao, Shanpeng Li, Haiping Duan, Shaojie Wang, Yongmei Liu, Ranran Du, Liang Cheng, Xiaocao Tian, Hua Zhang. **Licang CDC:** Dan Hu, Xiaoyan Zheng, Yujie Wang. **Heilongjiang Provincial CDC:** Wei Sun, Shichun Yan, Xiaoming Cui. **Nangang CDC:** Chi Wang, Zhenyuan Wu, Lishun Zhai, Zhaoxi Pang, Shiwen Dong. **Hainan Provincial CDC:** Huiming Luo, Jinyan Chen, Bin He, Dingwei Sun, Xingren Wang, Tingting Ou. **Meilan CDC:** Xiangyang Zheng, Dewei Zheng, Shuai Yang, Yilei Li, Lihui Li, Xingjiao Chen. **Jiangsu Provincial CDC:** Jinyi Zhou, Ran Tao, Jian Su, Xikang Fan, Zongming Cheng, Yuxiao Huang. **Suzhou CDC:** Yan Lu, Yujie Hua, Li Xing, Shuxian Wang, Jianrong Jin, Juping Ma, Jinchao Liu, Kaifei Zhu, Hongfu Ren, Xingfeng Shen. **Guangxi Provincial CDC:** Ge Zhong, Wei Mao, Zhenzhen Lu, Ling He. **Liuzhou CDC:** Lifang Zhou, Changping Xie, Jian Lan, Tingping Zhu, Jinxue Tan, Liuping Wei, Liyuan Zhou, Sisi Wang. **Sichuan Provincial CDC:** Xianping Wu, Ningmei Zhang, Xiaofang Chen, Xiaoyu Chang, Zhuo Wang, Yujin He. **Pengzhou CDC:** Mingqiang Yuan, Xia Wu, Xiaofang Chen, Zhaodong Wang, Qiang Sun, Yang Lin. **Gansu Provincial CDC:** Faqing Chen, Xiaolan Ren, Lijun Chang, Feiming Zhong. **Maiji CDC:** Jianjun Feng, Weijie Hu, Xiaofang Zhang, Yalin Chen, Fei Wang, Jun Wang. **Henan Provincial CDC:** Linqi Diao, Wanshen Guo, Zhiwei Han, Dongyang Zhao, Dengjun Zhu, Kai Kang, Shixian Feng, Huizi Tian, Yali Yan, Bing Han, Li Gao, Shaofang Li, Huafei Feng, Wei Tang. **Huixian CDC:** Xiaolin Li, Huarong Sun, Xiaocong Zhao, Ying Li, Chen Hu, Pan He, Xukui Zhang, Yuanyuan Jin, Hesheng Zhang. **Zhejiang Provincial CDC:** Min Yu, Ruying Hu, Hao Wang, Weiwei Gong, Jieming Zhong, Meng Wang, Chunxiao Xu, Keqing Gong. **Tongxiang CDC**: Hao Xu, Yuan Cao, Kaixu Xie, Lingli Chen, Xiaomei Tu,Chen Chen. **Hunan Provincial CDC:** Xiaojun Li, Li Yin, Huilin Liu, Yuan Liu, Yi Liu, Lei Yin, Xian Xie, Jing Wang. **Liuyang CDC:** Bo Xiao, Pingsheng Lou, Yuan Peng, Libo Zhang, Chan Qu, Qili Jiang, Yanling Chen, Yan Zhao.

Supplementary Methods

Measurement of clinical biochemistry

~18,000 participants were originally selected for a nested case-control study of stroke and of ischaemic heart disease [IHD] (at baseline all free of prior vascular disease and cancer, and not on statin therapy, with a censoring date of 1 January 2015, as described previously[1]). Incident intracerebral haemorrhage, ischaemic stroke, myocardial infarction (MI), and fatal IHD (non-MI) cases were selected from individuals with these events. Controls were selected from individuals who were free of the aforementioned events by the censoring date, frequency matched by age, sex, and area. Cases and controls were assayed for 17 plasma biochemistry measurements including urate level (μmol/L) at the Wolfson Laboratory (Clinical Trial Service Unit, Oxford, UK) using baseline plasma samples. Assays were performed using an AU680 clinical chemistry analyser (Beckman-Coulter, UK) except for plasma fibrinogen and cystatin C which were measured using a BN Prospec nephelometer analyser (Siemens, UK). All assays used standard manufacturers' reagents, calibrators, and settings. 16,817 participants had urate measurements.

Inverse probability of sampling weights

For analyses involving the biochemistry subset, inverse probability of sampling weights were applied to account for the inclusion/exclusion criteria and sampling scheme for the nested case-control study. Cases and controls were assigned different weights to reflect the different proportions of cases and controls from eligible participants in the entire China Kadoorie Biobank (CKB) cohort. The weights were calculated separately for controls and cases as the number of eligible participants divided by the number selected in the nested case-control study. The number and calculated weights for each case and control status are shown in **Table S5**. The weights were normalized (i.e. rescaled so that the sum of weights equals the total number of observations in the urate subset) and were applied in regression models for analyses involving urate level and hyperuricemia, where appropriate.

Covariates and model specification

Detailed baseline questionnaire assessment of socio-economic status, lifestyle factors (e.g. alcohol drinking, smoking, physical activity, diet including intake of meat, poultry, fish, fresh fruits, dairy products, soybean) and medical history factors, and physical measurements has been described previously[2-4]. Body mass index (BMI) was calculated from baseline anthropometry measurement as weight (kg) divided by the square of standing height (m^2). Mean systolic blood pressure (SBP) was calculated from the mean values of two measurements (and the last two readings of three measurements if inconsistent) measured using a UA-779 digital sphygmomanometer (A&D Instruments; Abingdon, UK) at baseline.[5]

Self-reported prior major diseases included: coronary heart disease, stroke or transient ischaemic attack, cancer, diabetes, tuberculosis, emphysema or bronchitis, liver cirrhosis or hepatitis, peptic ulcer, gallstone or gallbladder disease, kidney disease, rheumatoid arthritis, rheumatic heart disease, and fracture.

Unless otherwise specified, all regression models were (for Cox models) stratified by or (for logistic regression models) adjusted for sex and the ten study areas, and were further

adjusted for baseline age (continuous), education (four groups: no formal school, primary school, middle or high school, technical school/college or above), alcohol consumption (seven groups in men: non-drinkers, ex-drinkers, occasional, current [i.e. at least weekly] <140 g/week, 140-279 g/week, 280-419 g/week, 420+ g/week; four groups in women: non-drinkers, ex-drinkers, occasional, current), smoking status (five groups in men: never, occasional, ever regular <15, 15-24, ≥25 cigarettes equivalent/day; four groups in women: never, occasional, ex-regular, current), physical activity level (continuous, in metabolic equivalent of task per hour per day), fish or seafood intake (three groups: less than weekly, 1-3 days/week, 4-7 days/week), red meat intake (three groups: 1-3 days/week, 4-6 days/week, daily), poultry intake (three groups: never/rarely, monthly, at least weekly), soybean intake (three groups: less than weekly, 1-3 days/week, 4-7 days/week), dairy intake (three groups: never/rarely, monthly, at least weekly), and fresh fruit intake (three groups: less than weekly, 1-3 days/week, 4-7 days/week), and among the biochemistry subset time since last meal (i.e. fasting time before blood sample collection, continuous).

Confounding variables were selected based on a priori knowledge of standard socio-demographic, lifestyle, and cardiometabolic risk factors, and demonstrated associations with urate level, gout and major disease outcomes, and biochemistry measurement specific factors (i.e. fasting time before blood sample collection) where appropriate. There were no missing data for the key covariates included in the main analyses, whereas two individuals with missing BMI data were excluded from corresponding sensitivity analyses which involved BMI as a covariate.

As age was an important determinant of gout and multiple diseases of interest, all Cox models used age as the underlying time scale to appropriately account for age-related disease risks. Participants contributed time at risk from their age at baseline or 35 years (whichever later), with gout or gout duration (or exposure to major disease of interest) fitted as a time-updated exposure, and were followed up until the first occurrence of each type of disease outcome and were censored if they died of other causes, were lost to follow-up, or reached the general censoring date of 1 January 2019 or age 84 years, whichever occurred earlier.

Prospective associations of number of gout episodes with risks of comorbidities

Cox models were used to estimate adjusted HRs for major disease outcomes associated with number of gout episodes (none [reference group], 1 episode, 2+ episodes). Gout status was first derived as a time-updated variable, and among gout patients they were further categorised into having 1 episode or 2+ episodes with time exposed defined as starting from time at first gout episode. As for some gout patients the disease outcome of interest might have occurred between gout episodes, which may lead to reverse causation, sensitivity analyses were done by defining time exposed to start from the time at the last gout episode.

Prospective associations of co-morbidities (exposures) with risk of gout (outcome)

Cox models with a time-updated exposure for major disease of interest (see **Table S3** for detailed definitions of diseases), counting individuals with self-reported prior disease as exposed from baseline and among those without self-reported prior disease individuals with incident disease as exposed from their time of diagnosis, were used to estimate hazard ratios (HRs) for risk of gout associated with prevalent or incident co-morbidities and exposure duration preceding gout development.

Subgroup and sensitivity analyses

The prospective analyses of the associations of gout with disease risks were repeated by sex. For selected major disease outcomes (cardiovascular disease [CVD], chronic kidney disease [CKD], diabetes, arthropathies, all-cause mortality), analyses were conducted by subgroups defined by index age (i.e. age entering study [age at gout for gout patients]), study area, and education level.

Sensitivity analyses included:

(a) additional adjustment for further covariates (BMI [continuous, kg/m^2]), SBP [continuous, mmHg]) to assess independent associations beyond shared cardiometabolic risk factors;

(b) (a) plus excluding individuals with prior CVD and CKD;

(c) (b) plus excluding individuals with poor self-rated health at baseline; and

(d) delaying exposure by one year since the first gout episode to minimise reverse causation and impact from co-diagnosis of diseases (e.g. disease that was picked up during or shortly after gout-related hospitalisation, or vice versa).

Associations of urate level and hyperuricemia with risk of co-morbidities

Logistic regression models were used to estimate odds ratios (ORs) for co-morbidity outcomes (incident events over follow-up) associated with urate level (per standard deviation increment; in all participants with urate level measurements and in those without defined hyperuricemia, respectively) and hyperuricemia (yes/no), adjusted for major covariates and fasting time. Analyses for CVD, IHD and stroke were done using corresponding cases and controls of the nested case-control study. Analyses for non-CVD outcomes were done in all participants with measured urate level without self-reported prior major diseases, incorporating inverse probability weighting, with sensitivity analyses performed using robust standard errors. Dose-response associations were explored using restricted cubic splines with five knots at the 5th, 27.5th, 50th, 72.5th and 95th percentiles of the total distribution of urate level. Sensitivity analyses were performed using an alternative sex-specific definition of hyperuricemia (> 420 μmol/L in men, > 360 μmol/L in women).[6]

Figure S1. Map of the ten study sites of CKB

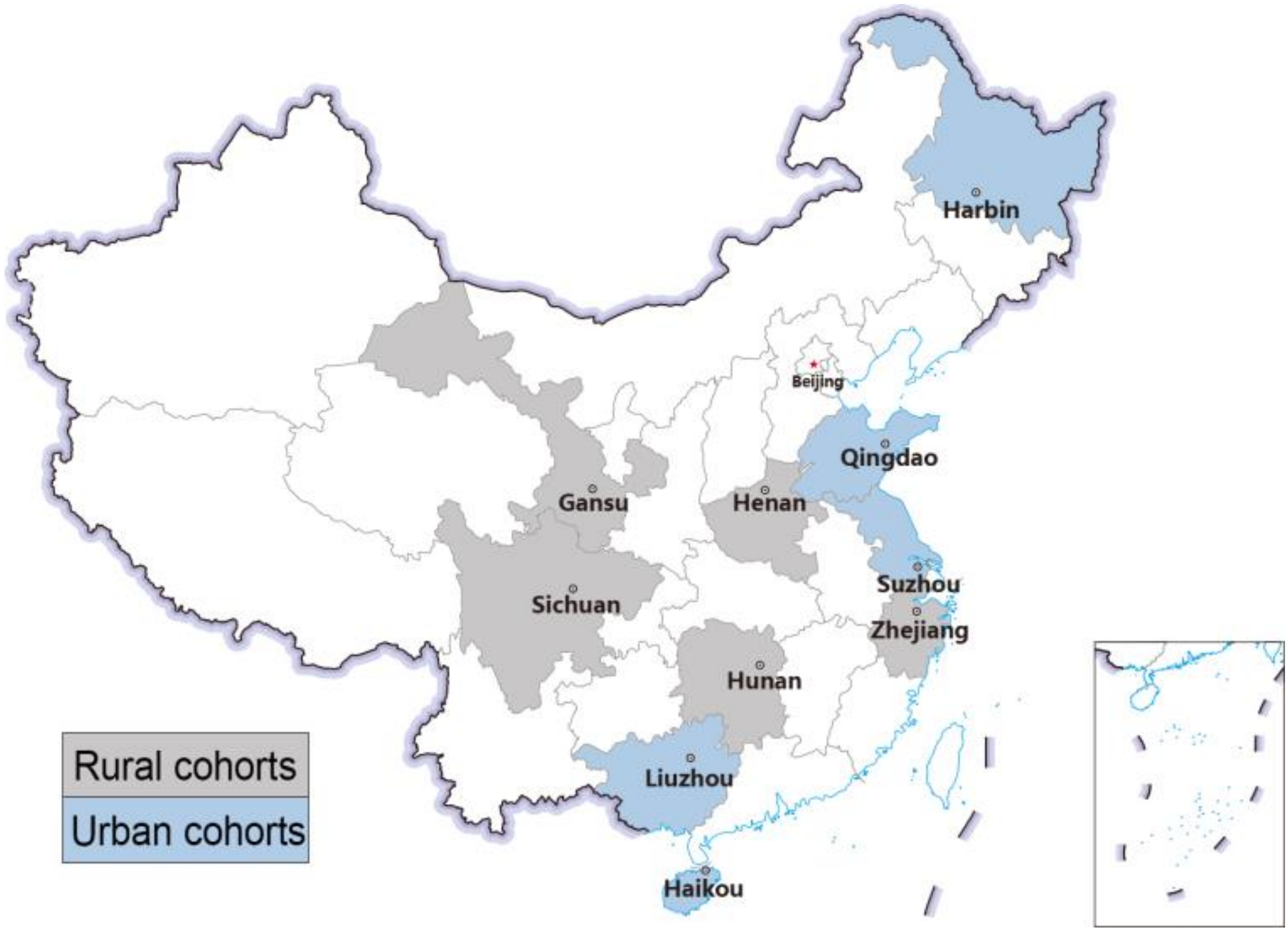

Table S1. Baseline characteristics of study participants by ten study sites of CKB

	Urban					Rural				
	Qingdao	Harbin	Haikou	Suzhou	Liuzhou	Sichuan	Gansu	Henan	Zhejiang	Hunan
**Demographic and lifestyle factors**										
Mean age, years	50.8	53.4	53.1	52.1	54.2	51.5	49.4	50.9	52.8	52.1
Women, %	56.0	59.6	63.6	58.0	61.5	61.7	61.3	56.1	58.4	56.0
Education > 6 years (i.e. primary school or above), %	76.8	85.8	65.8	37.7	75.1	34.7	26.9	51.3	19.7	36.3
Household income >20,000 yuan[a]/year, %	60.0	53.7	46.2	73.4	48.3	9.0	2.2	14.6	79.0	48.1
Current alcohol drinkers, %	22.0	22.7	6.0	17.4	11.8	23.1	3.0	11.8	16.8	11.3
Men	48.0	49.5	15.8	40.7	27.0	50.4	7.6	25.5	38.2	24.3
Women	1.5	4.5	0.4	0.6	2.3	6.2	0.1	1	1.5	1.2
Mean alcohol intake among current drinkers, g/week	264.6	180.2	205.9	291.2	178.3	383.5	200.8	238.2	342.4	267.9
Men	271.5	194.7	211.4	295.5	195.2	422.7	204.9	246.7	355.0	277.6
Women	89.3	72.6	79.4	79.4	53.4	187.2	60.2	66.8	108.7	109.5
Typically drink beer, % among current drinkers	41.6	51.9	14.8	7.7	12.8	1.3	10.9	10.7	10.3	3.3
Men	41.4	50.4	14.6	7.5	13	1.4	10.7	10.6	10.2	3.3
Women	47.7	63.3	20	14	10.9	0.9	16.7	13.2	12.4	2.3
Current smokers, %	26.3	24.0	15.3	28.9	19.5	31.6	28.3	25.9	27.6	31.1
Men	58.5	50.6	41.6	68.2	49.0	67.1	72.1	58.6	64.6	69.1
Women	1.1	6	0.3	0.4	1	9.5	0.6	0.3	1.2	1.2
Physical activity, mean MET-h/d	18.1	16.0	13.6	25.5	16.9	22.1	28.5	18.5	30.2	17.8
**Anthropometry**, mean										
Body mass index, kg/m^2	25.7	24.6	23.3	24.0	23.8	23.3	22.7	24.3	22.9	22.4
Systolic blood pressure, mmHg	132.0	128.0	124.4	132.8	128.2	129.2	131.4	134.1	135.9	131.4
Random glucose, mmol/Litre	6.4	6.4	6.2	6.1	6.4	6.2	5.9	5.9	5.6	5.9
**Medical history and health status[b]**, %										
Poor self-rated health	5.1	10.4	6.9	10.4	10.6	21.0	10.8	13.4	4.6	7.1
Coronary heart disease	5.1	9.4	1.4	1.1	4.6	0.5	1.7	2.1	0.8	3.3
Stroke or transient ischaemic attack	0.7	5.1	0.9	0.9	2.7	0.5	1.0	2.7	0.6	1.2
Kidney disease	0.3	4.8	0.8	0.7	0.4	4.4	0.9	0.3	0.7	0.7
Rheumatoid arthritis	1.2	3.3	3.4	2.1	2.7	2.5	1.8	1.4	1.4	1.4
Prevalent diabetes	9.6	10.3	7.0	5.3	8.4	4.0	3.4	4.7	5.0	3.5
**Frequent dietary consumption[c]**, %										
Red meat	72.8	59.8	86.1	44.7	86.6	42.5	17.0	4.3	48.3	43.4
Weekly poultry	35.6	29.2	41.2	45.0	82.1	12.1	1.1	3.6	42.3	6.2
Fish or seafood	22.7	1.4	62.6	17.6	3.2	0.2	0.1	0.2	3.2	8.4
Fresh fruits	63.0	59.5	27.6	28.8	52.9	21.5	16.9	4.7	17.5	7.3
Soybean products	13.0	17.2	4.9	8.0	10.4	2.4	3.8	3.6	23.0	10.6
Dairy products	38.6	33.5	8.5	9.4	21.3	3.8	6.7	4.3	1.1	1.7
Preserved vegetables	36.5	40.3	1.3	64.2	4.1	38.3	24.2	4.5	10.3	1.6
Spicy food	5.6	9.2	4.6	5.6	32.8	68.5	45.5	6.7	2.6	99.3

[a] At the exchange rate as of February 2025, 1 yuan is approximately equal to 0.14 U.S. dollars.
[b] Medical history and health status were self-reported at baseline, except for diabetes which was either self-reported or screen-detected.
[c] Frequent dietary consumption means 4+ days per week unless otherwise specified.
MET-h/d, metabolic equivalent of task per hour per day.

Table S2. Distribution of gout incidents (n=1402) in CKB by reporting source

Reporting source	N
Health insurance (i.e. inpatient hospitalisations)	1368
Death certificates	22
Disease registries	12

Table S3. Definitions of major diseases

	Self-reported medical history (i.e. prevalent cases)	ICD-10 code for incident cases over follow-up
Cardiovascular disease	Coronary heart disease, stroke or transient ischaemic attack	I00-I99
Ischaemic heart disease	Coronary heart disease	I20-I25
Stroke	Stroke or transient ischaemic attack	I60-I61, I63-I64, I69.0, I69.1, I69.3, I69.4
Cancer	Cancer	C00-C97
Chronic kidney disease	Kidney disease	E10.2+ , E11.2+, E12.2+, E13.2+, E14.2+, I12.0, I12.9, I13.0, I13.1, I13.2, I13.9, M10.3, M32.1+, N02, N03, N04, N05, N08.3*, N11, N12, N13, N15, N18, N19, N25, N26, N27.1, N27.9, N28.9, O10.2, O10.3, R94.4, T86.1, Z94.0
Urolithiasis[a]	--	N20-N23
Diabetes	Prevalent diabetes[b]	E10-E14
Chronic obstructive pulmonary disease	Emphysema or bronchitis	J41-J44
Liver disease	Liver cirrhosis or hepatitis	K70-K77
Diseases of oesophagus, stomach and duodenum	Peptic ulcer	K20-K31
Disorders of gallbladder, biliary tract and pancreas	Gallstone or gallbladder disease	K80-K87
Arthropathies excluding gout	Rheumatoid arthritis	M00-M09, M11-M25
Other musculoskeletal disorders	--	M30-M99
Infectious and parasitic diseases	Tuberculosis	A00-B99
Other autoimmune diseases	Rheumatic heart disease	D51.0, D51.9, D59.0, D59.1, D59.9, D69.0, D69.3, D86, E05.0, E05.5, E05.9, E06.3, E06.5, E06.9, E27.1, E27.2, E27.8, E27.9, E85, G35, G37, G36.0, H46, G61.0, G61.8, G61.9, G70.0, G70.8, G70.9, I00, I01, I02, I05, I06, I07, I08, I09, I73.0, J84.1, J84.8, J84.9, K50, K51, K52.3, K52.9, K90.0, K90.9, L10, L12, L40, L63, L80, L81.9, I52.8, I39, I41.8, G73.7, I32.8, G63.6, N08.5, N16.4, J99.1, H19.3
Fracture	Fracture	S02, S12, S22, S32, S42, S52, S62, S72, S82, S92, T02, T08, T10, T12
Multi-comorbidity (2+, 3+, 4+, or 5+)	Any 2 (or as indicated) or more of above, counting any cardiovascular disease as one disease	
All-cause mortality	--	Any coded or uncoded deaths

ICD-10, International Classification of Diseases, 10th Revision.

For analyses of prospective associations of gout with subsequent risks of incident diseases, participants with the relevant self-reported prior disease were excluded from the analyses (e.g. those with self-reported cancer were excluded from analyses for risk of incident cancer).

For analyses of prospective associations of major diseases with subsequent risk of gout, the exposure was defined as the presence of either the relevant self-reported prior disease or incident disease recorded during follow-up (e.g. either self-reported or incident cancer).

For analyses of associations of gout with multiple comorbidities, participants with any self-reported prior major disease were excluded from the analyses (see Supplementary Methods - Covariates and model specification for included diseases).

[a] Analyses of associations of gout with subsequent risk of urolithiasis excluded participants with prior self-reported kidney disease.

[b] Prevalent diabetes included both self-reported previously diagnosed diabetes and screen-detected diabetes by blood glucose test.[7]

Table S4. Number of disease events recorded via self-reported medical history at baseline and electronic health records over follow-up

	Self-reported medical history[a]			Cases recorded via EHR over follow-up[b]		
	Overall	No Gout	Gout cases	Overall	No Gout	Gout cases
Cardiovascular disease	23129	22998	131	150607	149768	839
Ischaemic heart disease	15472	15378	94	56510	56137	373
Stroke	8884	8838	46	67717	67349	368
Cancer	2578	2575	3	33730	33638	92
Chronic kidney disease	7574	7538	36	7972	7788	184
Urolithiasis	--	--	--	12504	12357	147
Diabetes	30300	30200	100	35563	35358	205
Chronic obstructive pulmonary disease	13288	13237	51	21669	21513	156
Liver diseases	6193	6177	16	6709	6666	43
Diseases of oesophagus, stomach and duodenum	20014	19926	88	31705	31487	218
Disorders of gallbladder, biliary tract and pancreas	30997	30888	109	20446	20372	74
Infectious and parasitic diseases	7659	7630	29	19137	18987	150
Arthropathies excluding gout[c]	10624	10554	70	14365	14080	285
Other musculoskeletal disorders[d]	--	--	--	45761	45428	333
Other autoimmune diseases	937	932	5	5921	5885	36
Fracture	35445	35312	133	17174	17089	85
**All-cause mortality**				56550	56324	226
Infectious diseases (ICD-10 code: A00-B99)				684 (1.2%)	682 (1.2%)	2 (0.9%)
Cancers (C00-C97)				17691 (31.3%)	17658 (31.4%)	33 (14.6%)
Cardiovascular diseases (I00-I99)				23290 (41.2%)	23193 (41.2%)	97 (42.9%)
Respiratory diseases (J00-J99)				5362 (9.5%)	5334 (9.5%)	28 (12.4%)
Digestive disease (K00-K93)				1183 (2.1%)	1172 (2.1%)	11 (4.9%)
Genitourinary diseases (N00-N99)				631 (1.1%)	616 (1.1%)	15 (6.6%)
Non-medical causes (S00-Y98)				3750 (6.6%)	3738 (6.6%)	12 (5.3%)
Any other causes				3959 (7.0%)	3931 (7.0%)	28 (12.4%)

[a] All were based on self-reported medical history at baseline questionnaire, except for prevalent diabetes which included both self-reported and screen-detected diabetes by blood glucose test.[7]
[b] Total number of cases recorded over follow-up time, including before and after gout diagnosis.
[c] For EHR-recorded incident arthropathies, 73% were arthrosis (ICD-10 code: M15-M19; 73% in non-gout and 68% gout participants) and 15% were rheumatoid arthritis (M05-M06; 15% in non-gout and 21% in gout patients). For self-reported medical history, cases were based on self-reported rheumatoid arthritis only.
[d] For EHR-recorded incident cases of other musculoskeletal disorders, 41% were cervical or other intervertebral disc disorders (ICD-10 code: M50-M51; 41% in non-gout and 36% gout participants), 39% were spondylopathies (M45-M49; 39% in non-gout and 38% in gout patients), and 4% were osteoporosis (M80-M81; 4% in non-gout and 6% in gout patients).
For mortality, number and proportion of major specific causes over all-cause mortality are presented.
EHR, electronic health records; ICD-10, International Classification of Diseases, 10th Revision.

Table S5. Number and weights for cases and controls in the subset of participants with urate measurement

Case-control status	N	Inverse probability weight
Control	6551	68.67
Intracerebral haemorrhage (ICD-10: I61, I69.1 – includes sequelae)	4079	1.36
Ischaemic stroke (ICD-10: I63, I69.3 – includes sequelae)	4660	5.49
Myocardial infarction (MI) (ICD-10: I21-I23 – includes sequelae)	1015	3.45
Fatal ischaemic heart disease (non-MI) (ICD-10: I20, I24, I25)	247	4.64
Subarachnoid stroke (ICD-10: I60, I69.0 – includes sequelae)	93	4.86
More than one event on the same day	172	1.87

In regression models, normalised weights (i.e. the weights were rescaled so that the sum of weights equals the total number of observations in the urate subset) were applied.
ICD-10, International Classification of Diseases, 10th Revision; MI, myocardial infarction.

Table S6. Adjusted ORs for having 2+ recorded gout episodes associated with socio-demographic factors, in 1402 gout patients

	Total N	N with 2+ gout episodes	Adjusted OR (95% CI)	P value
**Sex**				
Men	1071	303 (28%)	1.00	
Women	331	51 (15%)	0.43 (0.31-0.60)	<0.0001
**Region**				
Rural	700	194 (28%)	1.00	
Urban	702	160 (23%)	0.77 (0.39-1.52)	0.45
**Education**				
No formal	154	29 (19%)	1.00 (0.63-1.60)	
Primary	615	180 (29%)	1.25 (1.03-1.52)	
Middle/high	519	118 (23%)	0.90 (0.71-1.13)	
Technical school/College/University	114	27 (24%)	1.01 (0.62-1.62)	0.17
**Age at first recorded gout episode**				
<60 years	359	87 (24%)	1.00	
60+ years	1043	267 (26%)	0.81 (0.52-1.26)	0.35
Per 5 years older			0.47 (0.37-0.58)	<0.0001

CI, confidence interval; OR, odds ratio.

Logistic regression models were adjusted for sex, baseline age (continuous), and study area, where appropriate, selected as key socio-demographic determinants of health.

The distribution of number of gout episodes among 1402 gout patients were: N=1048 (75%) for 1 episode, N=206 (15%) for 2 episodes, and N=148 (10%) for 3 or more episodes. Among those with two or more episodes, the median duration between the first and second gout episode was 359 (interquartile range 52-813) days.

Table S7. Baseline characteristics of study participants with urate measurement

	Overall cohort (N=512,724)	Urate sample (N=16,817)
**Demographic and lifestyle factors**		
Mean age, years (SD)	52.0 (10.7)	56.7 (10.6)
Women, %	59.0	49.1
Urban, %	44.1	30.3
Education > 6 years (i.e. primary school or above), %	49.2	39.0
Household income > 20,000 yuan[a]/year, %	42.7	30.6
Current alcohol drinkers, %	14.9	16.5
Men	33.3	30.3
Women	2.1	2.3
Mean (SD) alcohol intake in current drinkers, g/week	271.7 (242.4)	287.8 (259.8)
Men	285.7 (245.3)	299.7 (263.2)
Women	115.6 (127.4)	123.9 (118.6)
Median (IQR) alcohol intake in current drinkers, g/week	195.0 (92.2-399.8)	230.1 (97.5-407.6)
Men	243.8 (110.6-407.6)	250.8 (115.1-407.6)
Women	67.9 (30.8-135.9)	83.6 (31.4-160.4)
Current smokers, %	26.4	33.6
Men	61.1	62.7
Women	2.4	3.5
Daily tea drinkers, %	26.1	28.8
Men	40.8	38.3
Women	15.9	19.0
Physical activity, mean MET-h/d (SD)	21.1 (13.9)	18.4 (13.3)
Physical activity, median MET-h/d (IQR)	17.5 (10.3-30.0)	14.3 (8.4-26.3)
**Anthropometry, mean (SD)**		
Body mass index, kg/m^2	23.7 (3.4)	23.6 (3.5)
Systolic blood pressure, mmHg	131.1 (21.3)	142.2 (25.6)
Random glucose, mmol/Litre	5.9 (1.9)	6.1 (2.4)
**Medical history and health status[b], %**		
Poor self-rated health	10.4	13.0
Cirrhosis or hepatitis	1.2	1.1
Emphysema or bronchitis	2.6	3.4
Peptic ulcer	3.9	4.0
Gallstone or gallbladder disease	6.0	5.5
Kidney disease	1.5	1.5
Rheumatoid arthritis	2.1	2.3
Prevalent diabetes	5.9	8.5
**Frequent dietary consumption[c], %**		
Red meat	47.2	37.9
Weekly poultry	28.2	18.3
Fish or seafood	8.9	4.9
Fresh fruits	28.2	19.9
Fresh vegetables	98.3	97.6
Soybean products	9.9	9.4
Dairy products	11.9	9.8
Preserved vegetables	22.6	20.4
Spicy food	30.1	36.5

Both mean (SD) and median (IQR) were reported for physical activity and weekly alcohol intake which had right-skewed distributions.
[a] At the exchange rate as of February 2025, 1 yuan is approximately equal to 0.14 U.S. dollars.
[b] Medical history and health status were self-reported at baseline, except for diabetes which was either self-reported or screen-detected.
[c] Frequent dietary consumption means 4+ days per week unless otherwise specified.
MET-h/d, metabolic equivalent of task per hour per day; SD, standard deviation; IQR, interquartile range.

Table S8. Mean urate level, proportion of hyperuricemia, and incidence rate of gout, overall and by demographic groups

	Urate subset						Total study population	
	Plasma urate level		Hyperuricemia (sex-specific cut-off)		Hyperuricemia (common cut-off)		Gout	
	N measured	Mean (95% CI), µmol/l	N	%	N	%	N events	Incidence rate (95% CI), per 100,000 person-years
**Overall (crude)**	16817	279.2[a]	1625	9.7[a]	2583	15.4[a]	1402	23.4
**Overall (IPW applied)**	16817	273.0	1625	7.9	2583	13.3	--	--
Sex								
Men	8560	304.7 (303.3-306.1)	1155	10.8 (10.2-11.4)	2113	21.4 (20.7-22.1)	1071	43.2 (40.6-45.8)
Women	8257	241.3 (239.9-242.7)	470	5.1 (4.6-5.7)	470	5.3 (4.6-6.0)	331	9.6 (8.6-10.6)
Area								
Rural	11724	264.8 (263.6-266.0)	819	6.2 (5.7-6.6)	1347	10.8 (10.2-11.4)	700	21.5 (19.9-23.1)
Urban	5093	301.6 (299.4-303.8)	806	14.0 (13.2-14.8)	1236	22.2 (21.1-23.2)	702	25.4 (23.5-27.3)
Age, years								
<45	2900	261.0 (258.8-263.3)	226	6.3 (5.4-7.2)	370	11.4 (10.3-12.5)	38	5.3 (3.6-7.0)
45-54	4287	268.4 (266.4-270.5)	337	6.3 (5.5-7.1)	553	11.0 (10.1-12.0)	187	11.0 (9.5-12.6)
55-64	5294	273.8 (271.9-275.7)	520	7.7 (7.0-8.5)	810	12.7 (11.8-13.6)	368	20.3 (18.2-22.4)
65-74	4128	284.4 (282.4-286.3)	514	10.6 (9.8-11.4)	807	17.3 (16.3-18.2)	431	34.0 (30.7-37.2)
75+	208	294.1 (285.7-302.5)	28	13.5 (10.1-16.8)	43	19.9 (15.9-24.0)	378	78.4 (70.2-86.6)
Highest education								
Primary school or below	10265	271.5 (270.2-272.8)	850	7.4 (6.9-7.9)	1331	12.9 (12.2-13.5)	769	22.6 (20.6-24.7)
Middle school	3942	273.3 (271.0-275.6)	433	8.7 (7.8-9.6)	686	13.4 (12.3-14.5)	331	22.4 (19.5-25.4)
High school or above	2610	281.5 (278.5-284.5)	342	9.6 (8.4-10.9)	566	15.9 (14.4-17.4)	302	22.1 (18.3-25.9)

Adjusted mean urate level and prevalence of hyperuricemia were estimated using multiple linear regression adjusted for sex, age (in ~10-year groups), ten study areas, and fasting time, as appropriate, with inverse probability weighting applied to account for the nested case-control study design.
Incidence rates were standardised by sex, age (in ~10-year groups) and area to the CKB study population as appropriate.
Age groups refer to age-at-risk for incidence rate, and baseline age for estimates of plasma urate level and hyperuricemia.
The sex-specific threshold for hyperuricemia was >420 µmol/L in men and >360 µmol/L in women, and the common threshold was >360 µmol/L.
[a] The corresponding mean urate level and proportion of hyperuricemia (sex-specific cut-off, and common cut-off) are 283.5 (SD 85.4) µmol/l and 10.9% and 16.8% in CVD cases, and 272.3 (SD 78.7) µmol/l and 7.7% and 13.1% in controls of the nested case-control study.
CI, confidence interval. IPW, inverse probability weighting.

Table S9. Associations of urate level and hyperuricemia with risk of gout

	SD of urate level, μmol/L	N event (exposed/ unexposed)	HR (95% CI)	P value
**Urate level, μmol/L**				
<300	**--**	8	1.00 (0.37-2.67)	
300-359	**--**	6	1.46 (0.53-4.02)	
360-419	**--**	9	3.01 (1.03-8.83)	
420-479	**--**	11	17.49 (7.85-38.98)	
480+	**--**	30	58.53 (33.07-103.60)	
**Per SD higher, overall**	**83.07**	**64**	2.99 (2.53-3.53)	**<0.0001**
Per SD higher, men	82.36	53	3.62 (2.94-4.46)	<0.0001
Per SD higher, women	68.76	11	1.67 (1.24-2.24)	0.0094
**Hyperuricemia, sex-specific threshold** (yes vs. no)				
**Overall**	**--**	**46/18**	14.39 (8.50-24.34)	**<0.0001**
Men	--	43/10	20.14 (10.51-38.57)	<0.0001
Women	--	3/8	4.86 (1.58-14.95)	0.055
**Hyperuricemia, common threshold** (yes vs. no)				
**Overall**	**--**	**50/14**	11.31 (6.30-20.33)	**<0.0001**
Men	--	47/6	15.76 (7.23-34.33)	<0.0001
Women	--	3/8	4.86 (1.58-14.95)	0.055

Cox models were stratified by sex (where appropriate) and ten study areas, and adjusted for baseline age, education and fasting time, with inverse probability weighting applied to account for the nested case-control study design. HRs associated with urate level groups were presented with group-specific 95% CIs to enable comparison between any two groups. The sex-specific threshold for hyperuricemia was >420 μmol/L in men and >360 μmol/L in women, and the common threshold was >360 μmol/L.
For conversion of HR to preferred unit scale: $HR_{per\ unit} = \exp(\ln(HR_{per\ SD}) * (unit/SD))$
SD, standard deviation; HR, hazard ratio; CI, confidence interval.

Figure S2. Age-specific mean urate level, hyperuricemia prevalence, and incidence rate of gout, by sex, with further adjustments

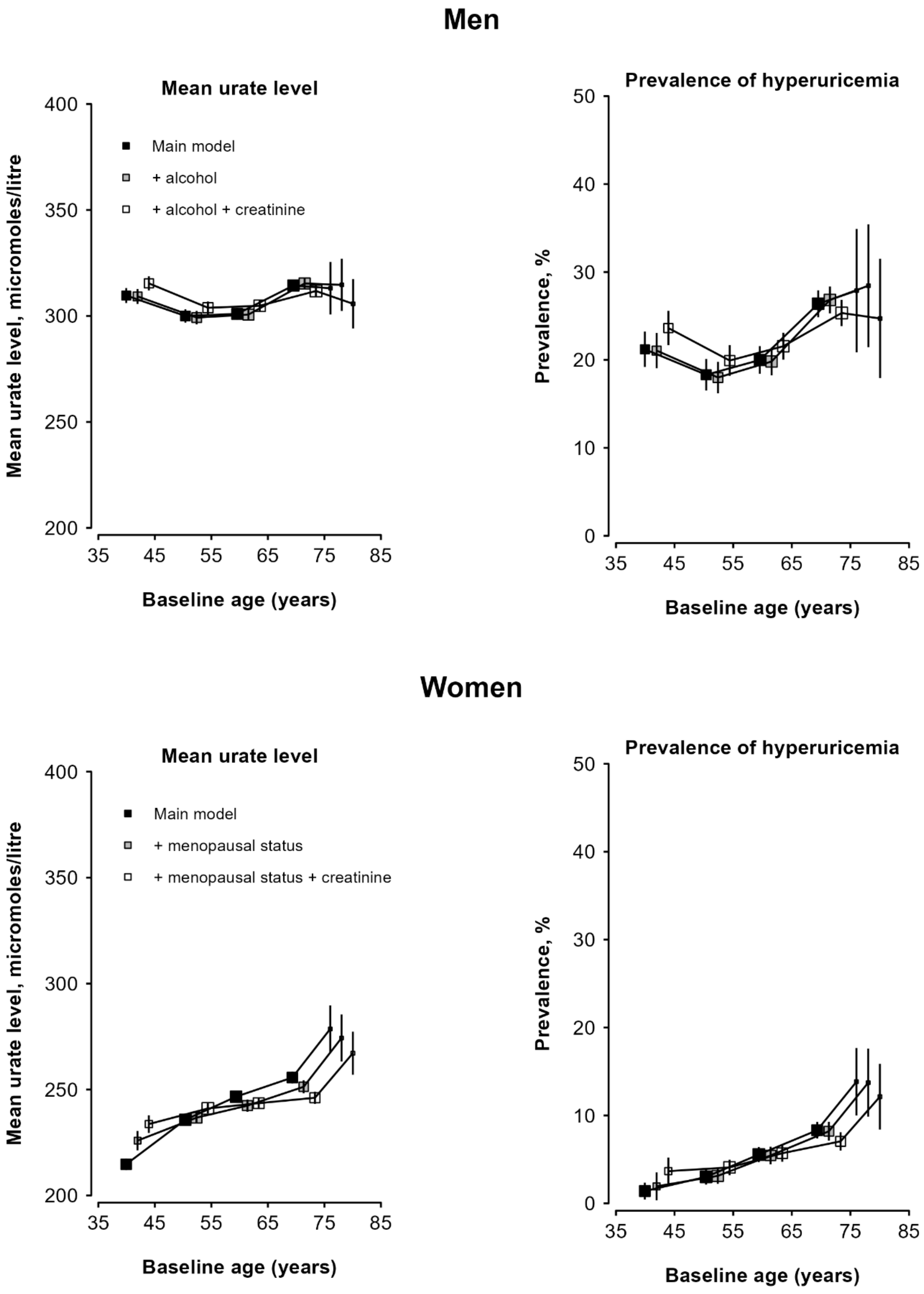

Adjusted mean urate level and prevalence of hyperuricemia were estimated using linear regression adjusted for study area and fasting time, with further adjustment for alcohol drinking status (for men), menopausal status (for women), and creatinine level. Each solid square represents the adjusted mean or prevalence with the area inversely proportional to the variance of the estimate. The error bars indicate 95% CIs. CI, confidence interval.

Figure S3. Age-specific mean urate level, hyperuricemia prevalence, and incidence rate of gout, by sex and study area

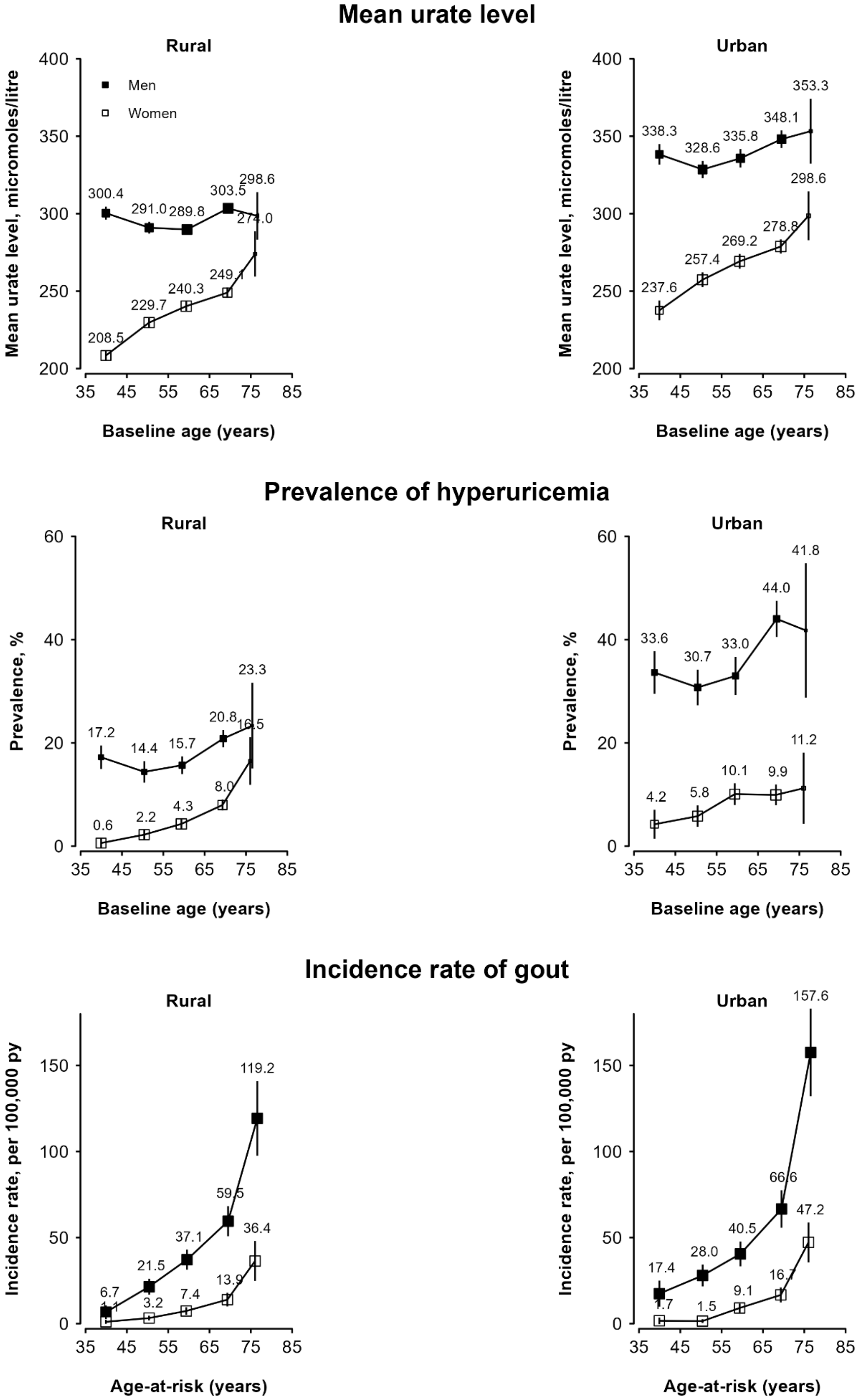

Adjusted mean urate level and prevalence of hyperuricemia were estimated using linear regression adjusted for study area and fasting time, with inverse probability weighting applied to account for the nested case-control study design. Incidence rates were standardised by study area to the CKB study population. Each solid square represents the adjusted mean, prevalence or incidence rate with the area inversely proportional to the variance of the estimate. The error bars indicate 95% CIs. CI, confidence interval.

Figure S4. Regional distribution of mean urate level, prevalence of hyperuricemia and incidence rate of gout, overall and by sex

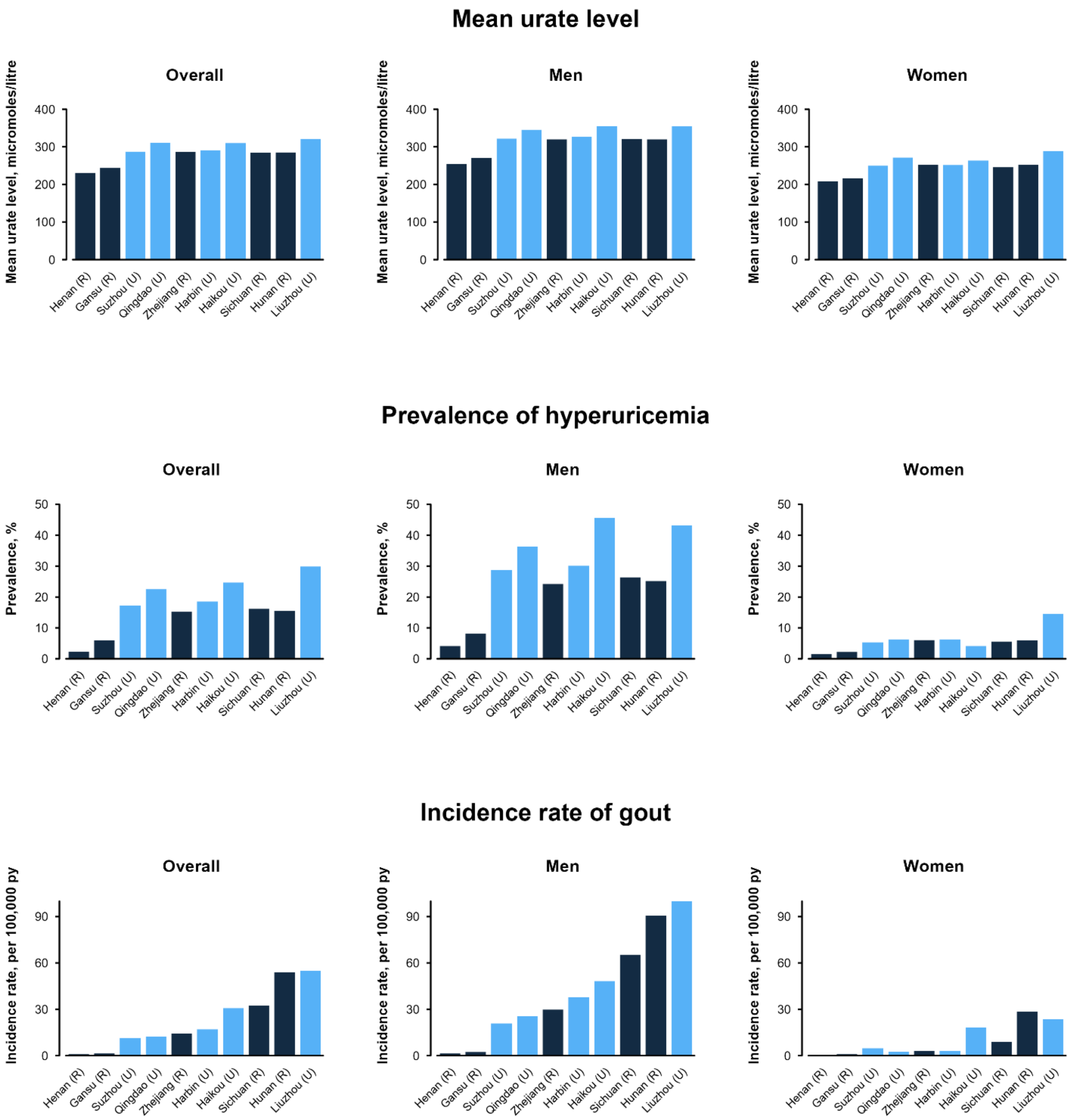

Adjusted mean urate level and prevalence of hyperuricemia were estimated using linear regression adjusted for age (in ~10-year groups), sex and fasting time, with inverse probability weighting applied to account for the nested case-control study design.
Incidence rates were standardised by age (in ~10-year groups) and sex to the CKB study population.
Light blue bars denote urban areas and dark blue bars denote rural areas (areas are ordered by overall gout incidence rate).

Figure S5. Comparison of prevalence of hyperuricemia using sex-specific cut-off and common cut-off

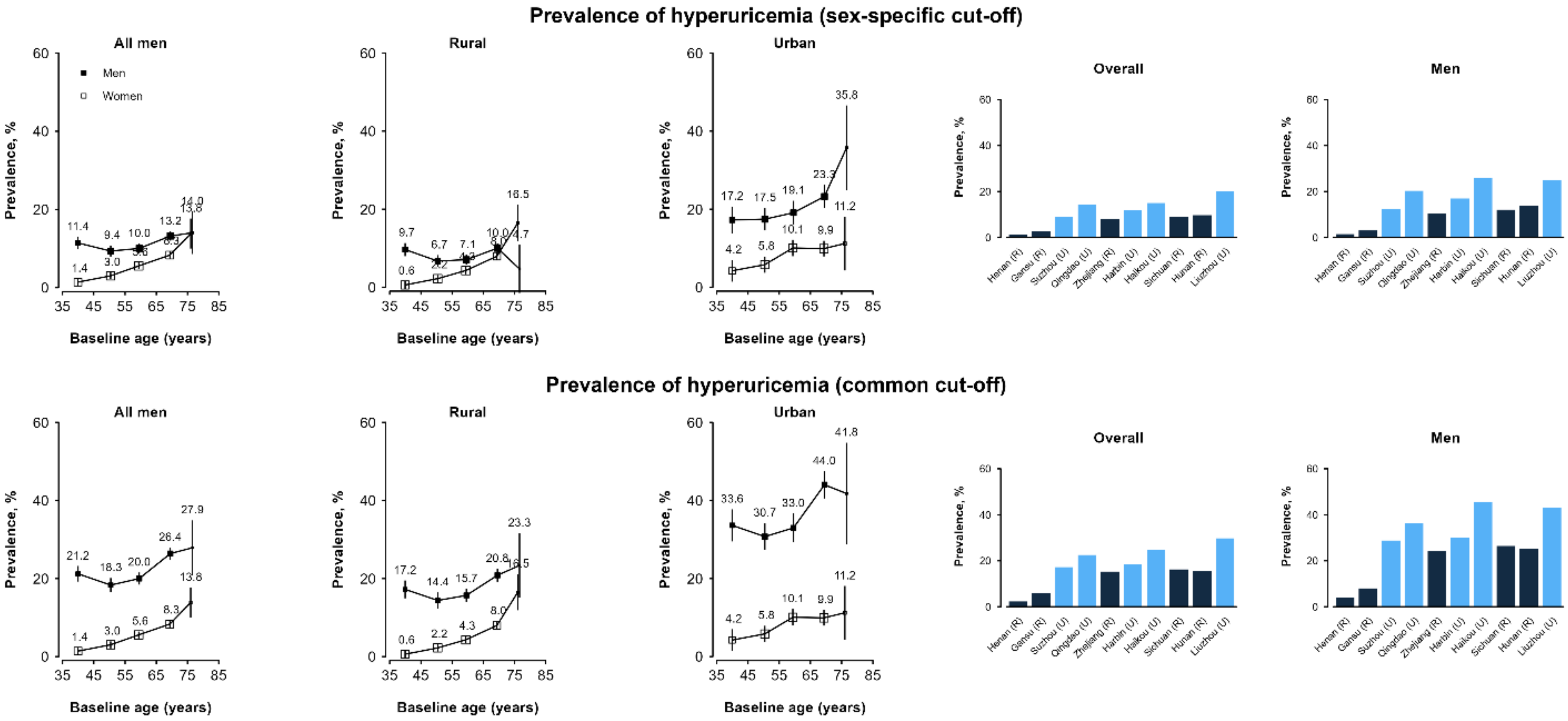

Adjusted prevalence of hyperuricemia were estimated using linear regression adjusted for age (in ~10-year groups), sex, study area and fasting time, where appropriate, with inverse probability weighting applied to account for the nested case-control study design.

The sex-specific threshold for hyperuricemia was >420 μmol/L in men and >360 μmol/L in women, and the common threshold was >360 μmol/L.

For line plots, each solid square represents the adjusted prevalence with the area inversely proportional to the variance of the estimate. The error bars indicate 95% CIs.

Light blue bars denote urban areas and dark blue bars denote rural areas (areas are ordered by overall gout incidence rate).

CI, confidence interval.

Figure S6. Associations of short-term gout duration with risks of major diseases and all-cause mortality

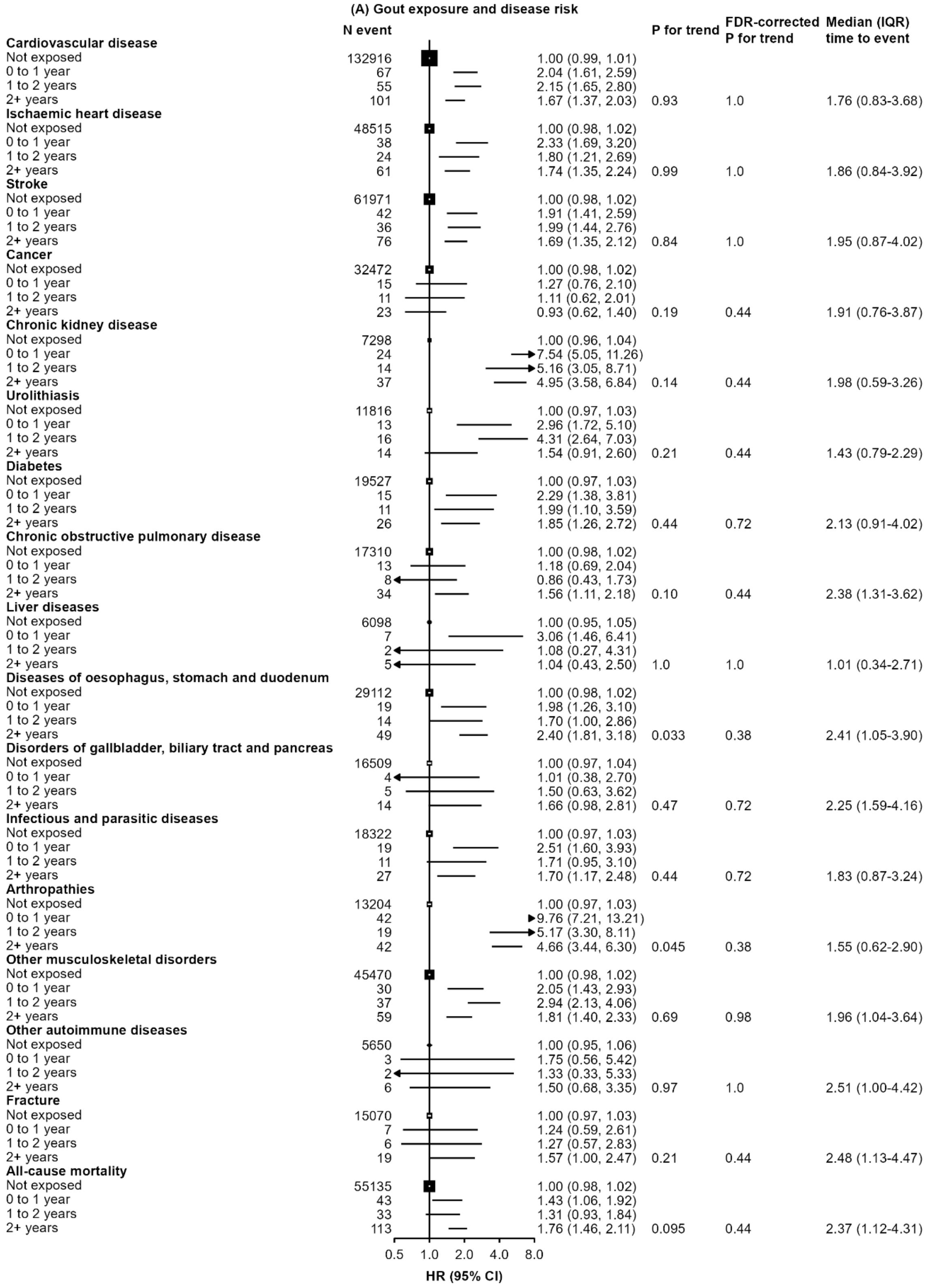

Cox models were stratified by sex and study areas and were adjusted for baseline age, education, smoking, alcohol, physical activity, fish intake, red meat intake, poultry intake, soybean intake, dairy intake, and fresh fruit intake. Test for trend was assessed among gout patients by fitting gout duration as an ordinal variable, Median time to event was calculated as the median time (years) from gout diagnosis to incident disease for each outcome. Each solid square represents HR with the area inversely proportional to the variance of the log hazard. The horizontal lines indicate group-specific 95% CIs. HR, hazard ratio; CI, confidence interval; FDR, false discovery rate; IQR, interquartile range

Figure S7. Associations of number of gout episodes with risks of subsequent major diseases and all-cause mortality

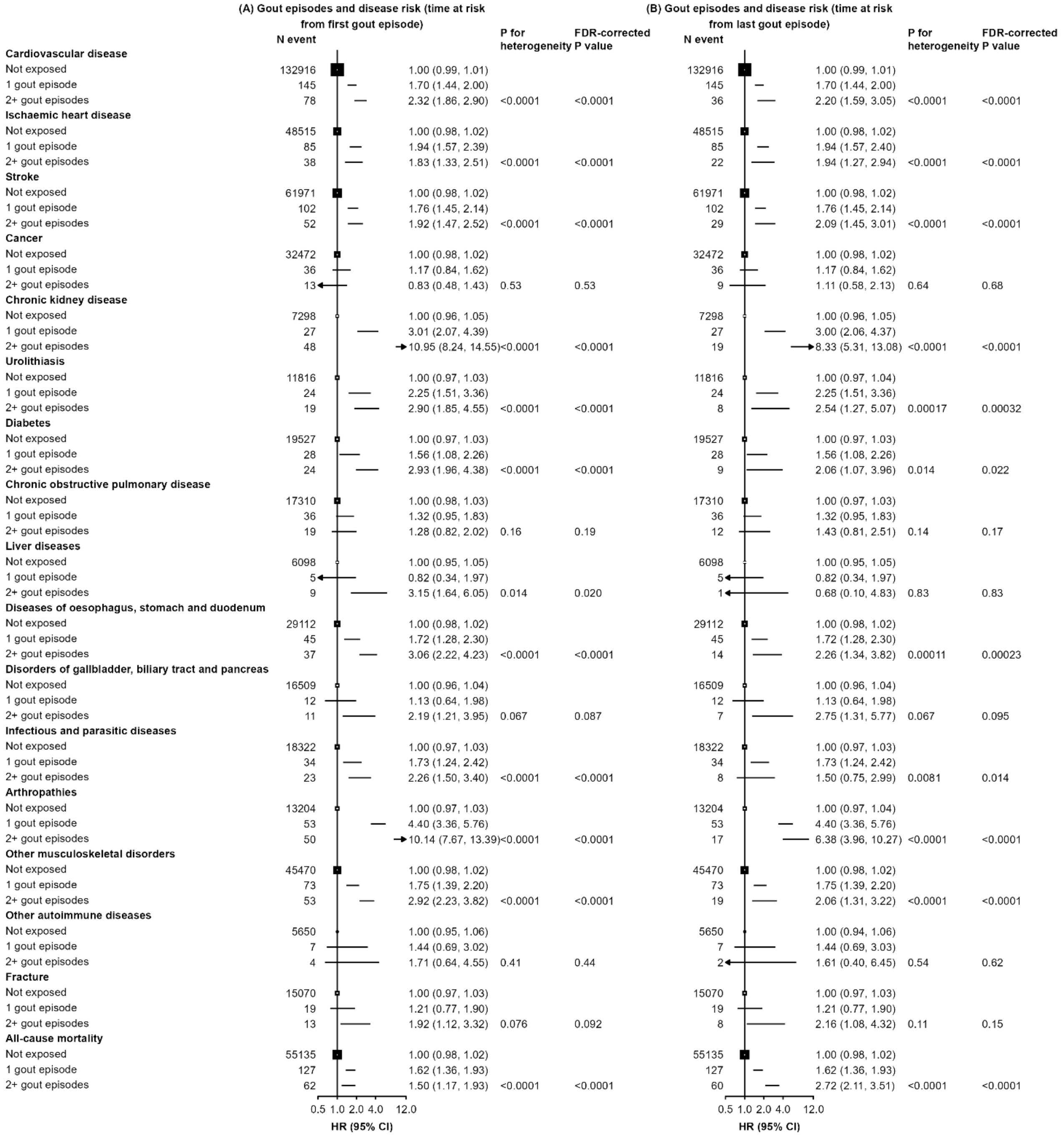

Cox models were stratified by sex and study areas and were adjusted for baseline age, education, smoking, alcohol, physical activity, fish intake, red meat intake, poultry intake, soybean intake, dairy intake, and fresh fruit intake. P for heterogeneity was obtained from a likelihood ratio test comparing Cox models with and without the categorical exposure variable (i.e. number of gout episodes). FDR-adjusted P values were applied to correct for multiple testing within (A) and (B), respectively. Conventions are as in Figure S6.

Figure S8. Associations of gout duration with risks of major diseases, and of preceding disease duration with subsequent gout risk

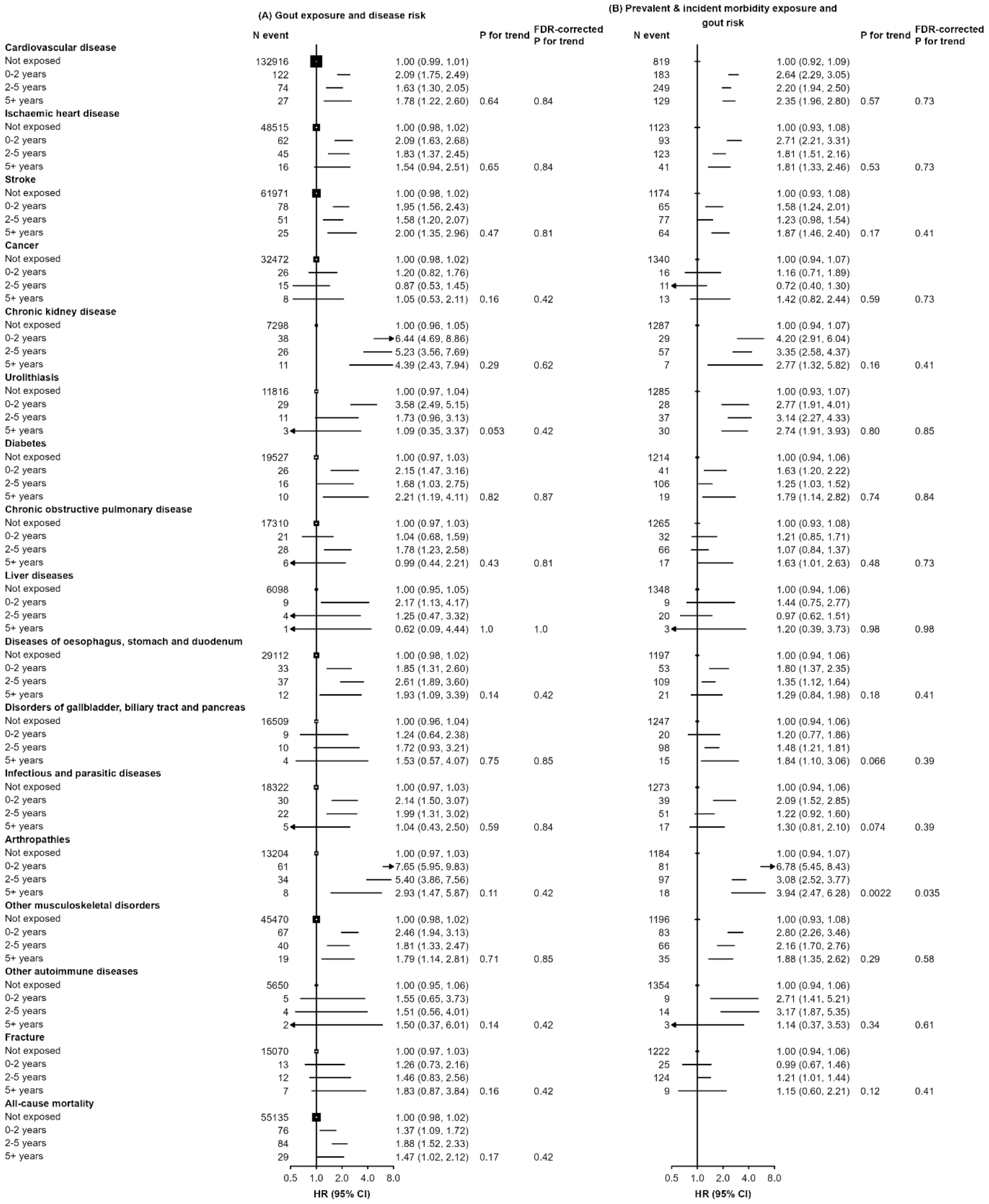

Cox models were stratified by sex and study areas and were adjusted for baseline age, education, smoking, alcohol, physical activity, fish intake, red meat intake, poultry intake, soybean intake, dairy intake, and fresh fruit intake. (A) displays HRs for risks of major diseases associated with gout duration, after excluding relevant prior diseases of interest. (B) displays HRs for risk of gout associated with duration of preceding prevalent and incident major diseases. FDR-adjusted P values were applied to correct for multiple testing within (A) and (B), respectively. Conventions are as in Figure S6.

Figure S9. Bi-directional associations of gout with major diseases, by sex

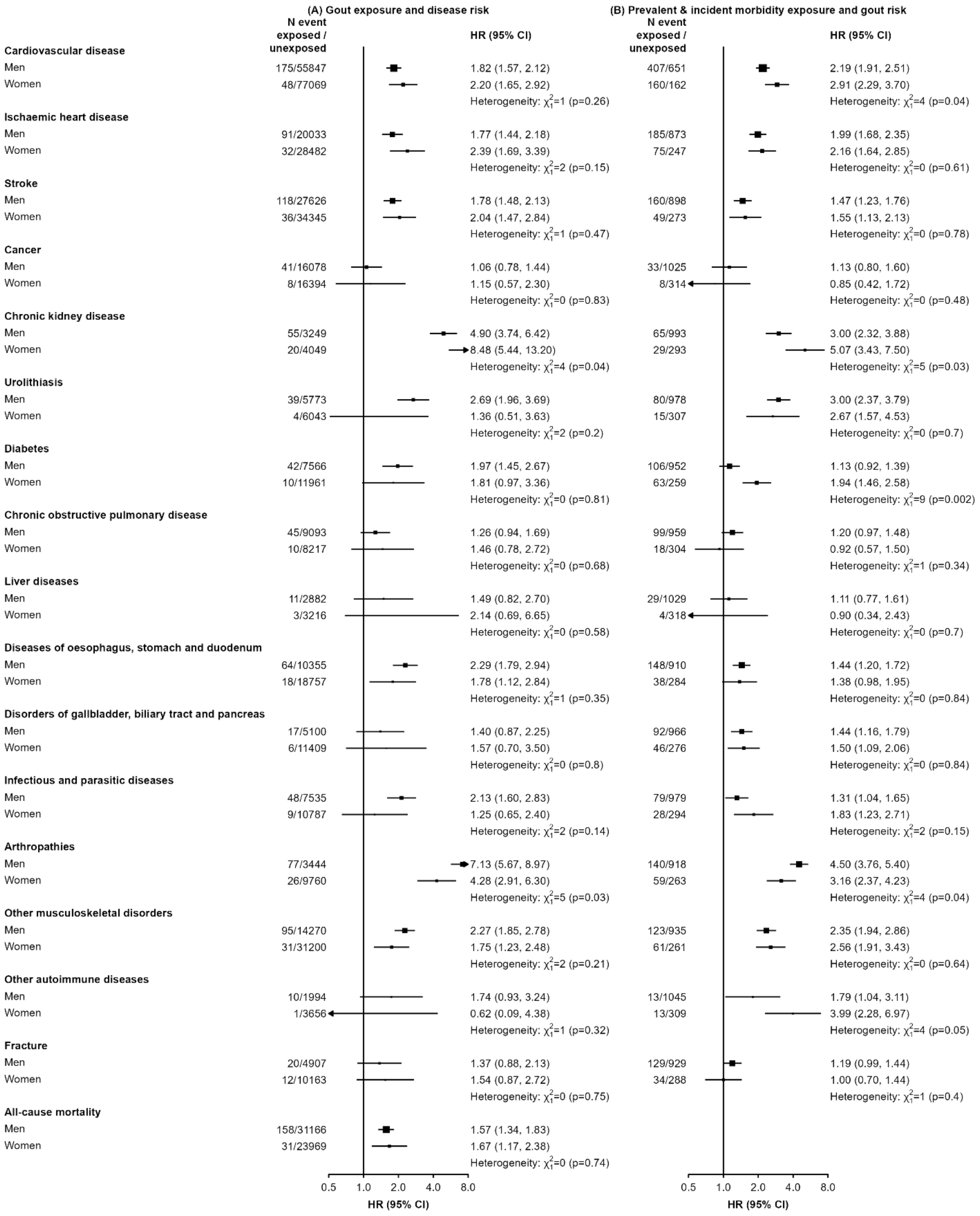

Cox models were stratified by study areas and were adjusted for baseline age, education, smoking, alcohol, physical activity, fish intake, red meat intake, poultry intake, soybean intake, dairy intake, and fresh fruit intake. (A) displays HRs for risks of major diseases associated with gout, after excluding relevant prior diseases of interest. (B) displays HRs for risk of gout associated with preceding prevalent and incident major diseases. Each solid square represents HR with the area inversely proportional to the variance of the log HR. The horizontal lines indicate 95% CIs. HR, hazard ratio; CI, confidence interval.

Figure S10. Sensitivity analyses of the associations of gout with risks of subsequent major diseases and all-cause mortality

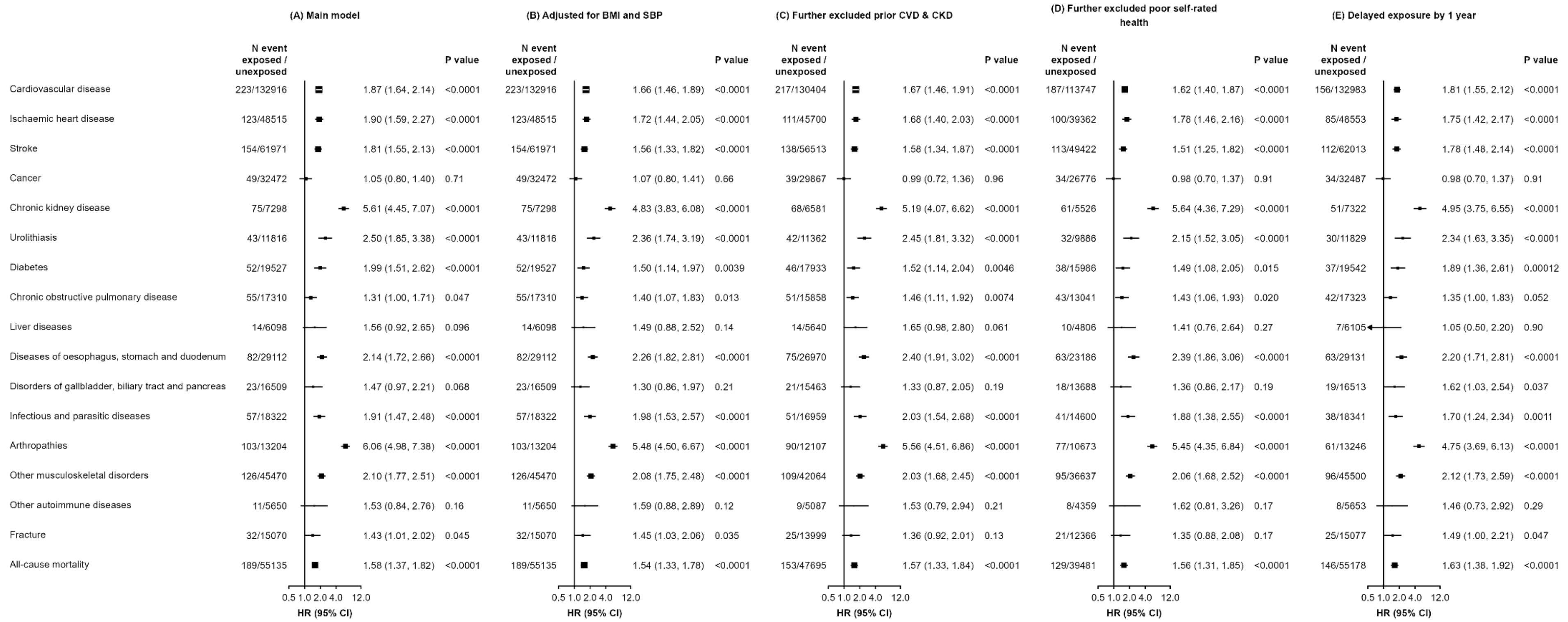

Cox models were stratified by sex and study areas and were adjusted for baseline age, education, smoking, alcohol, physical activity, fish intake, red meat intake, poultry intake, soybean intake, dairy intake, and fresh fruit intake. (A) displays the HRs for major diseases associated with gout from the main model, and with (B) additional adjustments, (C-D) additional adjustments plus further exclusions of self-reported prior disease or poor self-rated health, or (E) gout exposure modelled with delay by one year since the first gout episode, as indicated. Conventions are as in Figure S9.

Figure S11. Adjusted ORs for major diseases and all-cause mortality associated with urate level and hyperuricemia

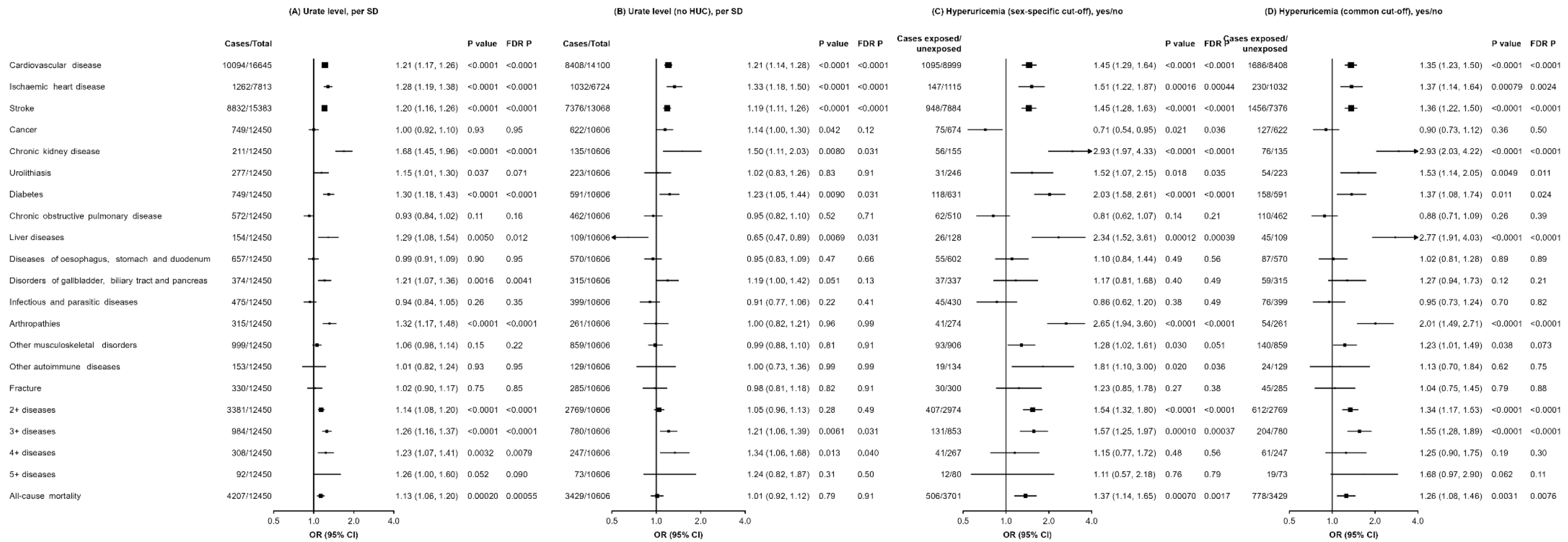

Logistic regression models were adjusted for sex, study areas, baseline age, fasting time, education, smoking, alcohol, physical activity, fish intake, red meat intake, poultry intake, soybean intake, dairy intake, and fresh fruit intake. For cardiovascular disease outcomes, analyses were conducted in corresponding cases and controls only. For non-cardiovascular disease outcomes, analyses were conducted in participants with urate level measurements excluding those with self-reported prior major diseases, with inverse probability weighting applied to account for the nested case-control study design. Analyses in (B) further restricted analyses to participants without defined hyperuricemia (common threshold). The sex-specific threshold for hyperuricemia in (C) was >420 μmol/L in men and >360 μmol/L in women, and the common threshold in (D) was >360 μmol/L. FDR-adjusted P values were applied to correct for multiple testing within each column, respectively. Each solid square represents OR with the area inversely proportional to the variance of the log OR. The horizontal lines indicate 95% CIs. OR, odds ratio; CI, confidence interval; HUC, hyperuricemia; FDR, false discovery rate.

Figure S12. Dose-response associations of urate level with major diseases and all-cause mortality

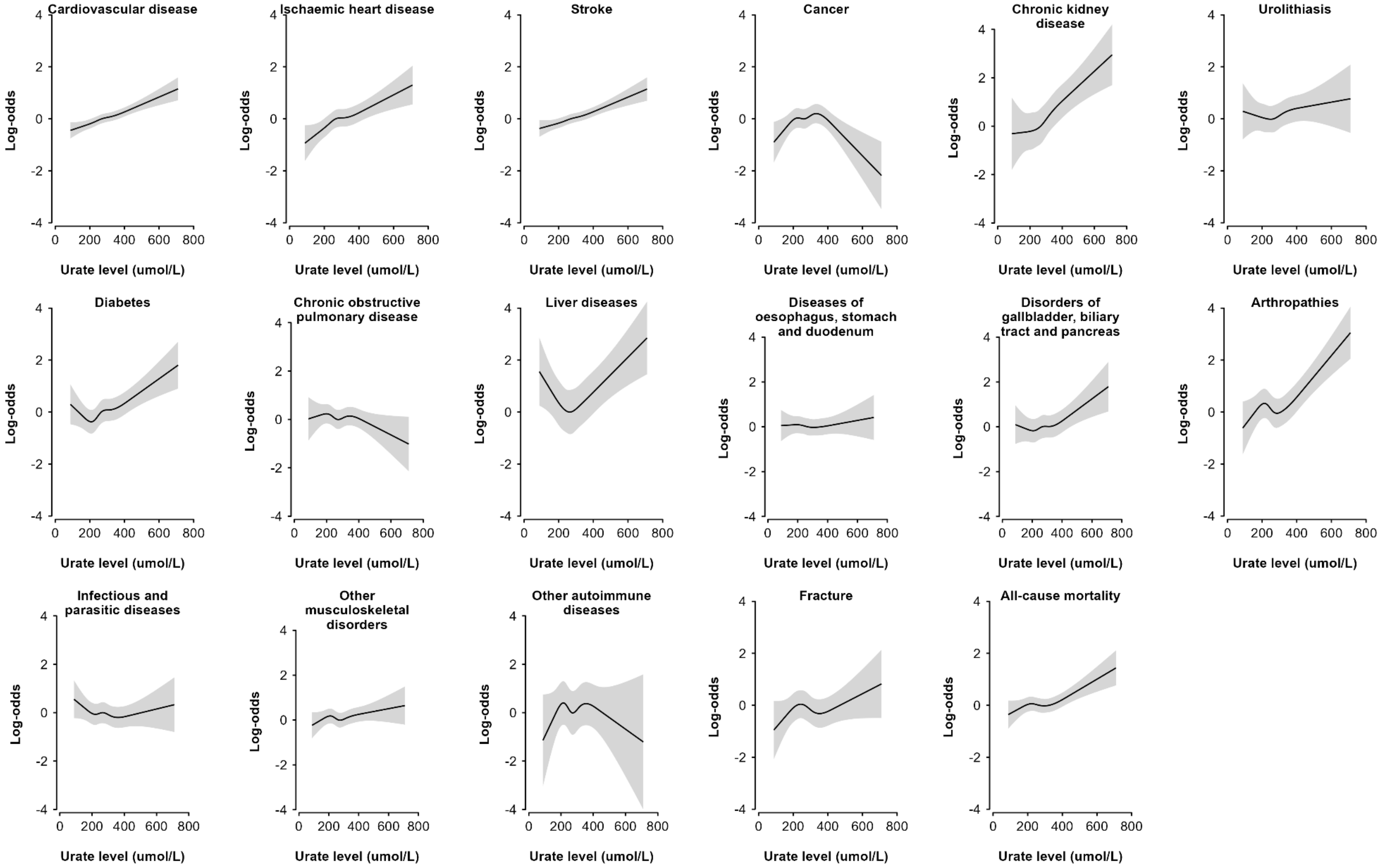

Logistic regression models were adjusted for sex, study areas, baseline age, fasting time, education, smoking, alcohol, physical activity, fish intake, red meat intake, poultry intake, soybean intake, dairy intake, and fresh fruit intake. For cardiovascular disease outcomes, analyses were conducted in corresponding cases and controls only. For non-cardiovascular disease outcomes, analyses were conducted in participants with urate level measurements excluding those with self-reported prior major diseases, with inverse probability weighting applied to account for the nested case-control study design. Restricted cubic splines were used with five knots at the 5th, 27.5th, 50th, 72.5th and 95th percentiles of the total distribution of urate level. The y-axis represents centered log-odds, calculated by subtracting the predicted log-odds at the median urate level from all predicted values, with the shaded area indicating the 95% confidence interval.

Figure S13. Adjusted ORs for major diseases and all-cause mortality associated with urate level and hyperuricemia, based on robust standard errors where applicable

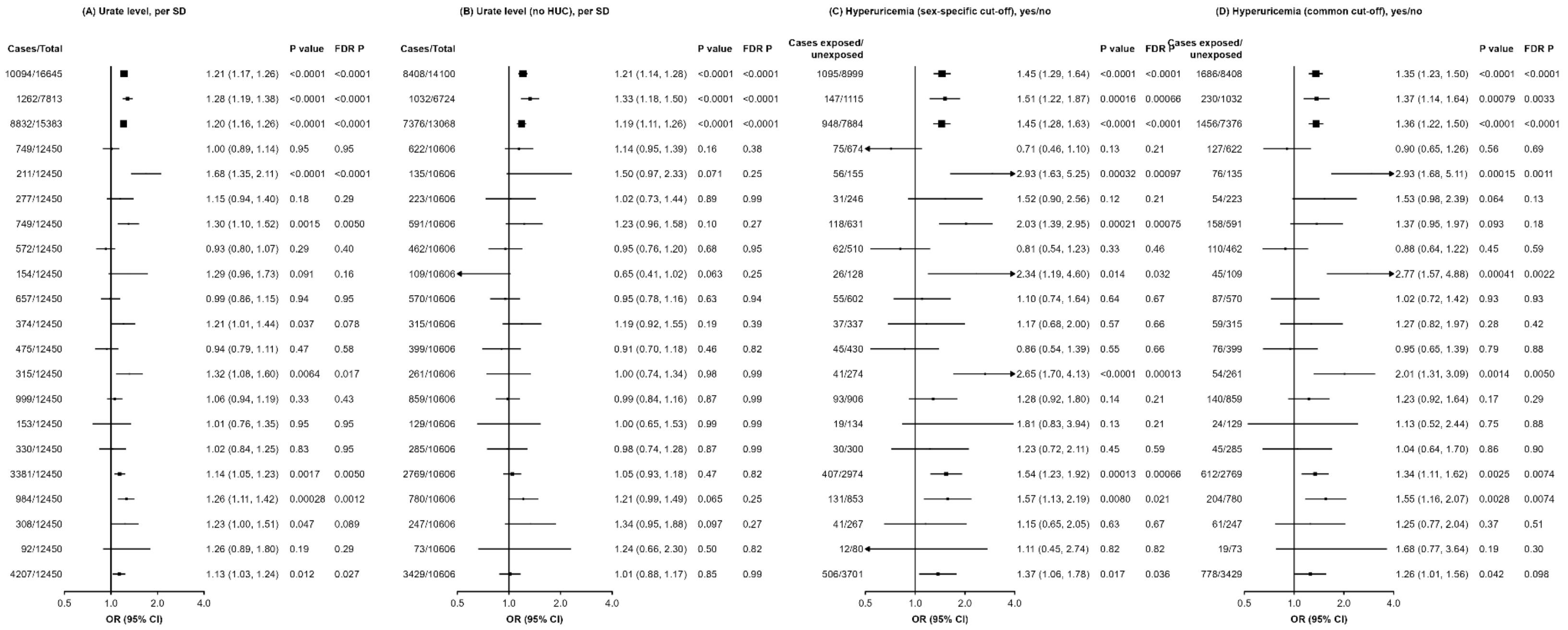

	(A) Urate level, per SD				(B) Urate level (no HUC), per SD			
	Cases/Total	OR (95% CI)	P value	FDR P	Cases/Total	OR (95% CI)	P value	FDR P
Cardiovascular disease	10094/16645	1.21 (1.17, 1.26)	<0.0001	<0.0001	8408/14100	1.21 (1.14, 1.28)	<0.0001	<0.0001
Ischaemic heart disease	1262/7813	1.28 (1.19, 1.38)	<0.0001	<0.0001	1032/6724	1.33 (1.18, 1.50)	<0.0001	<0.0001
Stroke	8832/15383	1.20 (1.16, 1.26)	<0.0001	<0.0001	7376/13068	1.19 (1.11, 1.26)	<0.0001	<0.0001
Cancer	749/12450	1.00 (0.89, 1.14)	0.95	0.95	622/10606	1.14 (0.95, 1.39)	0.16	0.38
Chronic kidney disease	211/12450	1.68 (1.35, 2.11)	<0.0001	<0.0001	135/10606	1.50 (0.97, 2.33)	0.071	0.25
Urolithiasis	277/12450	1.15 (0.94, 1.40)	0.18	0.29	223/10606	1.02 (0.73, 1.44)	0.89	0.99
Diabetes	749/12450	1.30 (1.10, 1.52)	0.0015	0.0050	591/10606	1.23 (0.96, 1.58)	0.10	0.27
Chronic obstructive pulmonary disease	572/12450	0.93 (0.80, 1.07)	0.29	0.40	462/10606	0.95 (0.76, 1.20)	0.68	0.95
Liver diseases	154/12450	1.29 (0.96, 1.73)	0.091	0.16	109/10606	0.65 (0.41, 1.02)	0.063	0.25
Diseases of oesophagus, stomach and duodenum	657/12450	0.99 (0.86, 1.15)	0.94	0.95	570/10606	0.95 (0.78, 1.16)	0.63	0.94
Disorders of gallbladder, biliary tract and pancreas	374/12450	1.21 (1.01, 1.44)	0.037	0.078	315/10606	1.19 (0.92, 1.55)	0.19	0.39
Infectious and parasitic diseases	475/12450	0.94 (0.79, 1.11)	0.47	0.58	399/10606	0.91 (0.70, 1.18)	0.46	0.82
Arthropathies	315/12450	1.32 (1.08, 1.60)	0.0064	0.017	261/10606	1.00 (0.74, 1.34)	0.98	0.99
Other musculoskeletal disorders	999/12450	1.06 (0.94, 1.19)	0.33	0.43	859/10606	0.99 (0.84, 1.16)	0.87	0.99
Other autoimmune diseases	153/12450	1.01 (0.76, 1.35)	0.95	0.95	129/10606	1.00 (0.65, 1.53)	0.99	0.99
Fracture	330/12450	1.02 (0.84, 1.25)	0.83	0.95	285/10606	0.98 (0.74, 1.28)	0.87	0.99
2+ diseases	3381/12450	1.14 (1.05, 1.23)	0.0017	0.0050	2769/10606	1.05 (0.93, 1.18)	0.47	0.82
3+ diseases	984/12450	1.26 (1.11, 1.42)	0.00028	0.0012	780/10606	1.21 (0.99, 1.49)	0.065	0.25
4+ diseases	308/12450	1.23 (1.00, 1.51)	0.047	0.089	247/10606	1.34 (0.95, 1.88)	0.097	0.27
5+ diseases	92/12450	1.26 (0.89, 1.80)	0.19	0.29	73/10606	1.24 (0.66, 2.30)	0.50	0.82
All-cause mortality	4207/12450	1.13 (1.03, 1.24)	0.012	0.027	3429/10606	1.01 (0.88, 1.17)	0.85	0.99

	(C) Hyperuricemia (sex-specific cut-off), yes/no				(D) Hyperuricemia (common cut-off), yes/no			
	Cases exposed/ unexposed	OR (95% CI)	P value	FDR P	Cases exposed/ unexposed	OR (95% CI)	P value	FDR P
Cardiovascular disease	1095/8999	1.45 (1.29, 1.64)	<0.0001	<0.0001	1686/8408	1.35 (1.23, 1.50)	<0.0001	<0.0001
Ischaemic heart disease	147/1115	1.51 (1.22, 1.87)	0.00016	0.00066	230/1032	1.37 (1.14, 1.64)	0.00079	0.0033
Stroke	948/7884	1.45 (1.28, 1.63)	<0.0001	<0.0001	1456/7376	1.36 (1.22, 1.50)	<0.0001	<0.0001
Cancer	75/674	0.71 (0.46, 1.10)	0.13	0.21	127/622	0.90 (0.65, 1.26)	0.56	0.69
Chronic kidney disease	56/155	2.93 (1.63, 5.25)	0.00032	0.00097	76/135	2.93 (1.68, 5.11)	0.00015	0.0011
Urolithiasis	31/246	1.52 (0.90, 2.56)	0.12	0.21	54/223	1.53 (0.98, 2.39)	0.064	0.13
Diabetes	118/631	2.03 (1.39, 2.95)	0.00021	0.00075	158/591	1.37 (0.95, 1.97)	0.093	0.18
Chronic obstructive pulmonary disease	62/510	0.81 (0.54, 1.23)	0.33	0.46	110/462	0.88 (0.64, 1.22)	0.45	0.59
Liver diseases	26/128	2.34 (1.19, 4.60)	0.014	0.032	45/109	2.77 (1.57, 4.88)	0.00041	0.0022
Diseases of oesophagus, stomach and duodenum	55/602	1.10 (0.74, 1.64)	0.64	0.67	87/570	1.02 (0.72, 1.42)	0.93	0.93
Disorders of gallbladder, biliary tract and pancreas	37/337	1.17 (0.68, 2.00)	0.57	0.66	59/315	1.27 (0.82, 1.97)	0.28	0.42
Infectious and parasitic diseases	45/430	0.86 (0.54, 1.39)	0.55	0.66	76/399	0.95 (0.65, 1.39)	0.79	0.88
Arthropathies	41/274	2.65 (1.70, 4.13)	<0.0001	0.00013	54/261	2.01 (1.31, 3.09)	0.0014	0.0050
Other musculoskeletal disorders	93/906	1.28 (0.92, 1.80)	0.14	0.21	140/859	1.23 (0.92, 1.64)	0.17	0.29
Other autoimmune diseases	19/134	1.81 (0.83, 3.94)	0.13	0.21	24/129	1.13 (0.52, 2.44)	0.75	0.88
Fracture	30/300	1.23 (0.72, 2.11)	0.45	0.59	45/285	1.04 (0.64, 1.70)	0.86	0.90
2+ diseases	407/2974	1.54 (1.23, 1.92)	0.00013	0.00066	612/2769	1.34 (1.11, 1.62)	0.0025	0.0074
3+ diseases	131/853	1.57 (1.13, 2.19)	0.0080	0.021	204/780	1.55 (1.16, 2.07)	0.0028	0.0074
4+ diseases	41/267	1.15 (0.65, 2.05)	0.63	0.67	61/247	1.25 (0.77, 2.04)	0.37	0.51
5+ diseases	12/80	1.11 (0.45, 2.74)	0.82	0.82	19/73	1.68 (0.77, 3.64)	0.19	0.30
All-cause mortality	506/3701	1.37 (1.06, 1.78)	0.017	0.036	778/3429	1.26 (1.01, 1.56)	0.042	0.098

Logistic regression models were adjusted for sex, study areas, baseline age, fasting time, education, smoking, alcohol, physical activity, fish intake, red meat intake, poultry intake, soybean intake, dairy intake, and fresh fruit intake. For cardiovascular disease outcomes, analyses were conducted in corresponding cases and controls only. For non-cardiovascular disease outcomes, analyses were conducted in participants with urate level measurements excluding those with self-reported prior major diseases, with inverse probability weighting applied to account for the nested case-control study design and robust standard errors used. Analyses in (B) further restricted analyses to participants without defined hyperuricemia (common threshold). The sex-specific threshold for hyperuricemia in (C) was >420 μmol/L in men and >360 μmol/L in women, and the common threshold in (D) was >360 μmol/L. FDR-adjusted P values were applied to correct for multiple testing within each column, respectively. Conventions are as in Figure S11.

Figure S14. Adjusted ORs for having multiple comorbidities associated with gout, overall and by sex

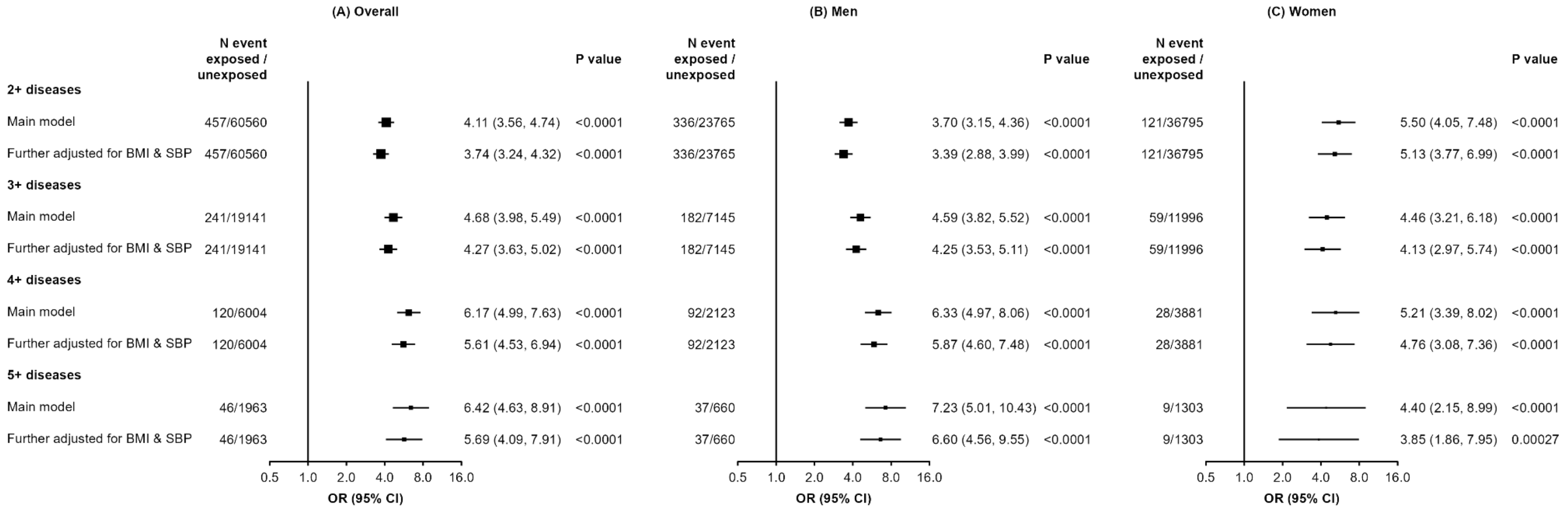

Logistic regression models were adjusted where appropriate for sex, study areas, baseline age, education, smoking, alcohol, physical activity, fish intake, red meat intake, poultry intake, soybean intake, dairy intake, and fresh fruit intake. Participants with self-reported prior major diseases were excluded. Each solid square represents OR with the area inversely proportional to the variance of the log OR. The horizontal lines indicate 95% CIs. OR, odds ratio; CI, confidence interval.

Figure S15. Total expected hospitalisations overall and by major disease categories, and Kaplan-Meier curves for overall survival, from age-at-risk of 35 years, by gout status

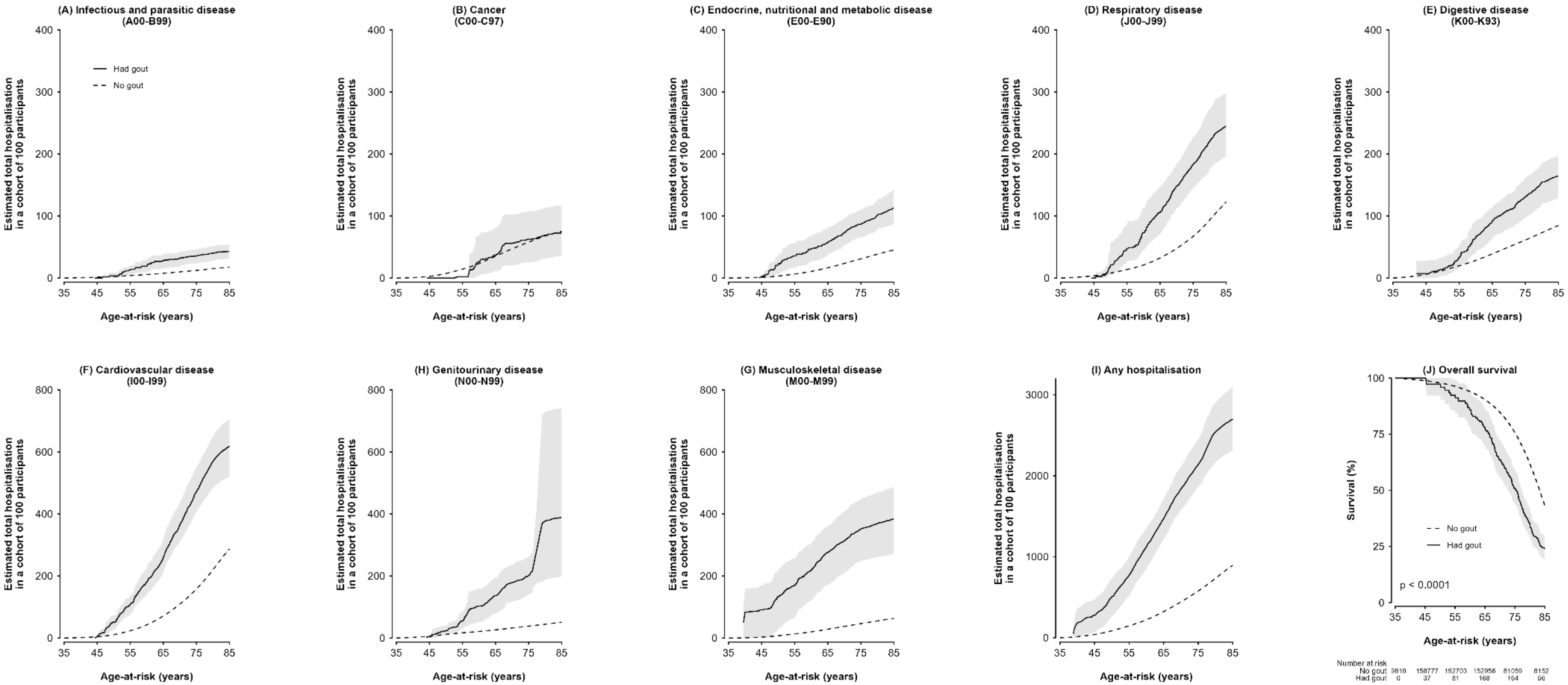

Grey bands show 95% confidence intervals. (A) to (I) showed total expected hospitalisations overall and by major disease categories, and (J) showed Kaplan-Meier curves for overall survival, by gout status. For (J): Numbers at risk shown for age 35, 45, 55, 65, 75, and 85 years; P-value for Cox model score (logrank) test. ICD-10, International Classification of Diseases, 10th Revision.

References

1. Pang Y, Kartsonaki C, Du H, et al. Physical Activity, Sedentary Leisure Time, Circulating Metabolic Markers, and Risk of Major Vascular Diseases. *Circulation Genomic and precision medicine* 2019; **12**(9): 386-96.

2. Millwood IY, Li L, Smith M, et al. Alcohol consumption in 0.5 million people from 10 diverse regions of China: prevalence, patterns and socio-demographic and health-related correlates. *International journal of epidemiology* 2013; **42**(3): 816-27.

3. Im PK, Millwood IY, Guo Y, et al. Patterns and trends of alcohol consumption in rural and urban areas of China: findings from the China Kadoorie Biobank. *BMC public health* 2019; **19**(1): 217.

4. Im PK, Millwood IY, Chen Y, et al. Problem drinking, wellbeing and mortality risk in Chinese men: findings from the China Kadoorie Biobank. *Addiction* 2020; **115**(5): 850-62.

5. Clarke R, Wright N, Walters R, et al. Genetically Predicted Differences in Systolic Blood Pressure and Risk of Cardiovascular and Noncardiovascular Diseases: A Mendelian Randomization Study in Chinese Adults. *Hypertension* 2023; **80**(3): 566-76.

6. Li Q, Li X, Wang J, et al. Diagnosis and treatment for hyperuricemia and gout: a systematic review of clinical practice guidelines and consensus statements. *BMJ Open* 2019; **9**(8): e026677.

7. Bragg F, Holmes MV, Iona A, et al. Association between diabetes and cause-specific mortality in rural and urban areas of China. *JAMA* 2017; **317**(3): 280-9.
